# Dimeric Rh Complexes Supported by a Bridging Phosphido/Bis(Phosphine) PPP Ligand

**DOI:** 10.1021/acs.organomet.3c00492

**Published:** 2024-04-22

**Authors:** Mario N. Cosio, Samuel R. Lee, Qingheng Lai, Nattamai Bhuvanesh, Jia Zhou, Oleg V. Ozerov

**Affiliations:** † Department of Chemistry, Texas A&M University, College Station, Texas 77842, United States; ∥ State Key Laboratory of Urban Water Resource and Environment, School of Science, Harbin Institute of Technology, Shenzhen 518055, China

## Abstract

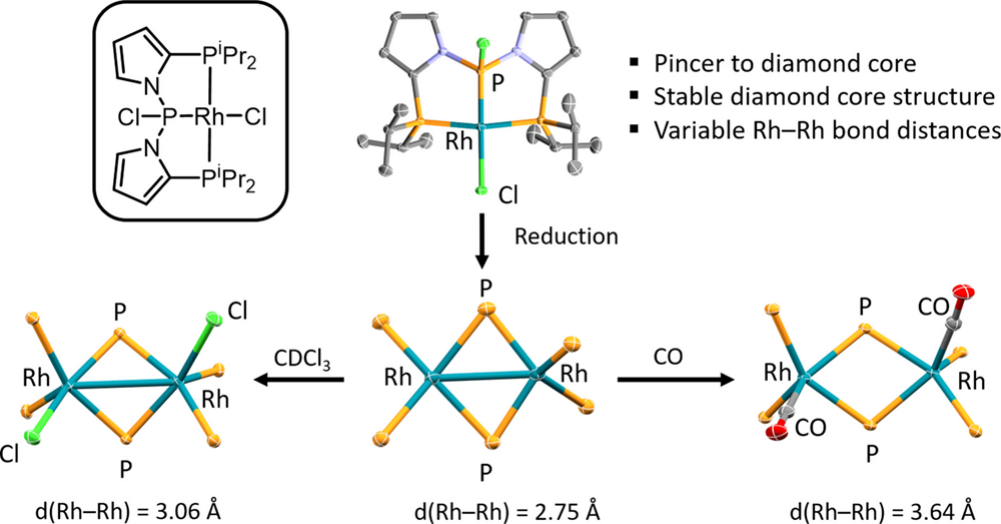

Rh complexes of a tridentate PPP ligand bearing 1,2-pyrrolediyl linkers have been prepared, including examples with the central P donor being either a phosphine or a phosphide. Three bimetallic Rh complexes containing the diamandoid Rh_2_P_2_ core (P = phosphido) have been structurally and spectroscopically characterized. The Rh–Rh interaction in these three dimers was examined by way of structural comparisons and DFT investigations.

## Introduction

Pincer ligands have cemented an important place in organometallic chemistry as a versatile class of ancillary ligands for transition metals.^1−6^ Pincers are tridentate meridional ligands, and their construction can be generally thought of as three donor atoms connected via two linkers. The 1,2-phenylene linker has become rather ubiquitous,^6^ possessing rigidity and the “correct” spacing for preorganizing the pincer structure to comfortably bind most transition metals.

**Figure 1:**
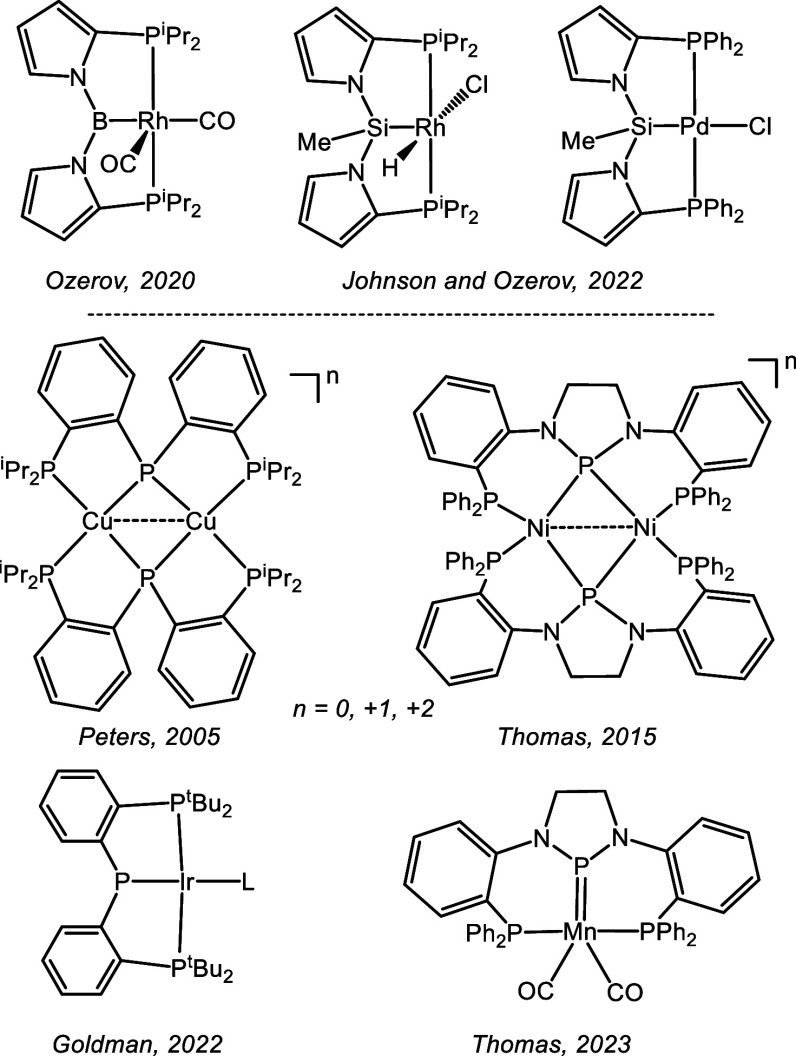
Selected examples of complexes of pincer ligands containing 1,2-pyrrolediyl linkers (top) and of complexes of phosphide/bis(phosphine) PPP pincer ligands (bottom).

We recently became interested in using 1,2-pyrrolediyl as an alternative linker and reported complexes of PBP and PAlP pincer ligands,^7^ as well as PSiP ligands jointly with the Johnson group.^8^ Here, we report on the synthesis of a pyrrole-derived PPP pincer and its Rh complexes. The phosphido/bis(phosphine) PPP ligand with a 1,2-phenylene linker has been pioneered by Peters with a few transition metals (Figure 1).^9,10^ Most recently, the Goldman group showed that (PPP^tBu^)Ir complexes are remarkably active catalysts for transfer alkane dehydrogenation.^11,12^ The structurally closest PPP relative comes from the Thomas group (Figure 1), where the central phosphide site is also connected to two nitrogens.^13,14^ The PPP ligands in the complexes in Figure 1 have been known to form both monomeric complexes with a meridionally binding PPP pincer and dimeric complexes with bridging phosphides, presaging the observations in this work.

## Results and Discussion

### Synthesis and Characterization of (PP^Cl^P)RhCl

**Scheme 1:**
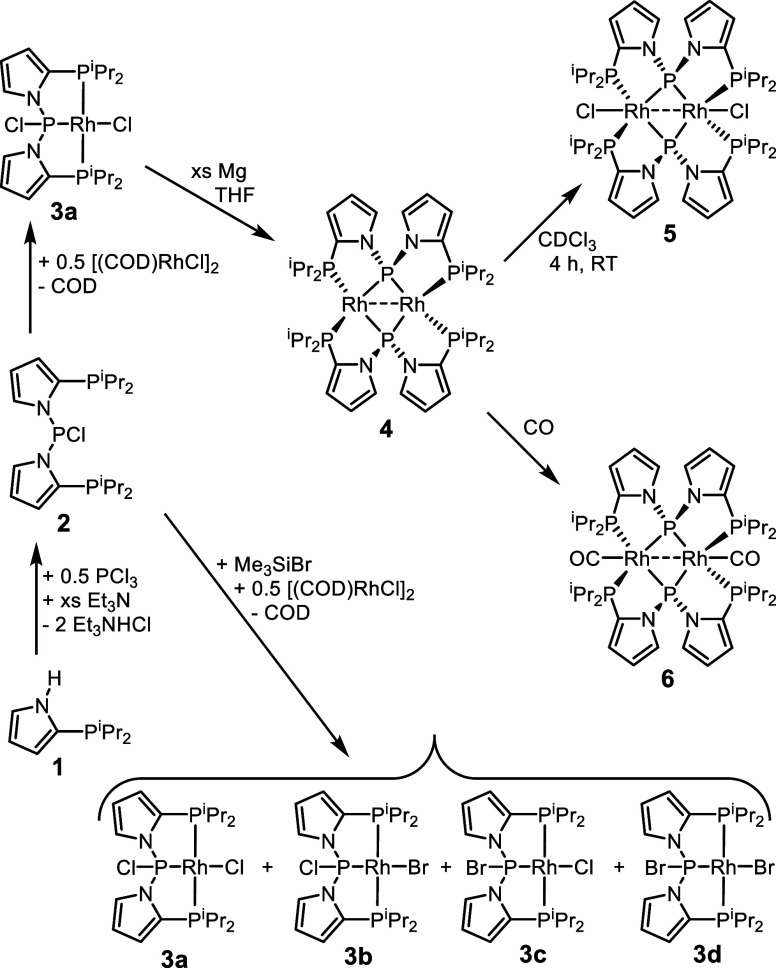
Synthesis of the PPP Ligand 2 and Its Rh Complexes

We previously reported the synthesis of 2-diisopropylphosphinopyrrole (**1**), a convenient ligand building block.^15^ The acidity of the pyrrolic N–H allows for straightforward attachment of this N to the main group elements. Indeed, the installation of two 2-phosphinopyrrolyl substituents onto the central phosphorus atom proceeded smoothly via reaction of **1** with 0.5 equiv PCl_3_ in the presence of 6 equiv of Et_3_N (Scheme 1). Upon workup, a decent (47%) yield of new tris(phosphine) **2** was obtained. Compound **2** reacted with 0.5 equiv of [(COD)RhCl]_2_ in <15 min at RT to give a 90% isolated yield of the Rh complex **3a**. When **1** was treated with 1 equiv of Me_3_SiBr prior to the introduction of 0.5 equiv of [(COD)RhCl]_2_, Cl/Br exchange at both the P-Hal and Rh-Hal positions took place,^16^ and a mixture of four compounds **3a**–**d** was observed by ^31^P NMR spectroscopy. Each of the four compounds gave rise to a similar pattern in the ^31^P{^1^H} NMR spectrum, consistent with a monomeric structure. Most tellingly, there were two resonances in each ^31^P{^1^H} NMR spectrum in a 2:1 ratio, which displayed coupling to each other (^2^*J*_P–P_ = 40–44 Hz) as well as to ^103^Rh (^1^*J*_P–Rh_ = 122–124 and 285–291 Hz). The smaller ^1^*J*_P–Rh_ value belongs to the outer -PPr^i^_2_ arms and is generally in the range observed for (PXP)Rh pincer complexes with similar substituents on P.^7,8,17−19^ The greater ^1^*J*_P–Rh_ value corresponds to the central P atom, which bears three heteroatoms and is *trans* to a halide. The chemical shifts of the ^31^P NMR resonances of the −PPr^i^_2_ arms in **3a**–**d** fall within the narrow range of 37.7–38.2 ppm. For the central phosphine signals, the ones bearing Cl are farther downfield (105.2 ppm for **3a** and 104.9 ppm for **3b**) than the ones bearing Br (84.8 ppm for **3c** and 84.4 ppm for **3d**).

**Figure 2:**
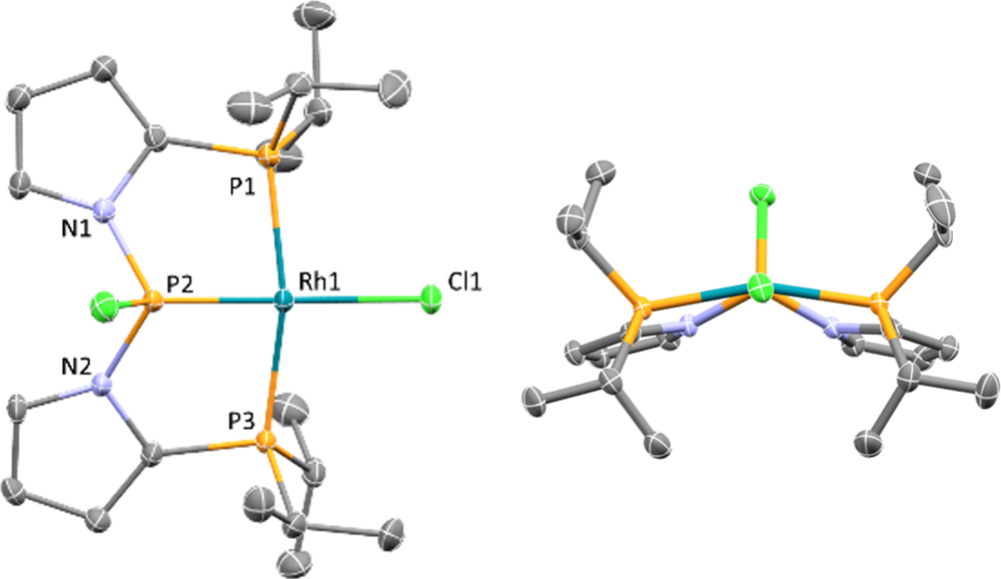
ORTEP drawing (50% probability ellipsoids) of **3a**. H atoms and solvent fluorobenzene are omitted for clarity. Selected distances (Å): Rh1–P1: 2.2977(8), Rh1–P2: 2.099(1), Rh1–P3: 2.3013(7), Rh1–Cl: 2.377(1), P2–N1: 1.714(3), and P2–N2: 1.708(2). (left) top-down view (right) view down the P–Rh–Cl axis.

The structure of **3a** suggested by the NMR data was confirmed in the solid state by an X-ray diffraction (XRD) study (Figure 2). Compound **3a** adopts an approximately square-planar geometry about Rh. The central P–Rh–Cl vector is nearly perfectly linear, while the *trans* P–Rh–P angle is only about 159°, a consequence of the chelate constraint. There is a stark, ca. 0.2 Å difference between the central Rh–P distance and the Rh–P distances associated with the outer phosphine arms. The short Rh–P distance to the central P is primarily owing to the electron-withdrawing nature of the substituents on it. For comparison, the Rh–P distances in (acac)Rh(CO)(P(N-pyrrolyl)_3_),^20^ [(F_3_P)_2_RhCl]_2_,^21^ and *trans*-ClRh(PPh_3_)_2_(P(OPh)_3_)^22^ are ca. 2.17, 2.12, and 2.14 Å, respectively. Trends in the M–P distances as a function of having a carbon vs more electronegative substituents on P have been discussed elsewhere.^23−25^

### Synthesis and Spectroscopic Characterization of Dirhodium Complexes

Reduction of **3a** with Mg metal in THF furnished **4** as a dark-brown solid in a good (74%) yield after workup. Dissolution of **4** in CDCl_3_ led to the change in color to dark blue over the period of 4 h. Compound **5** was isolated from this solution by crystallization at low temperature in 50% yield. Formation of **5** likely proceeds via chlorine atom transfer from CDCl_3_.^26^ Exposure of **4** to a CO atmosphere resulted in the rapid formation of the new product **6**, isolated as a brown powder.

Each of the three dimeric compounds **4**–**6** presented NMR spectra with resonances in the expected range for diamagnetic complexes, including ^31^P NMR resonances. The ^1^H, ^13^C, and ^31^P NMR spectra of **4** at ambient temperature were consistent with the time-averaged *C*_*2*_ symmetry for the dimeric molecule. The bridging phosphido ^31^P{^1^H} NMR resonance appeared as a triplet of triplets from the coupling to two ^31^P nuclei (^2^*J*_P–P_ = 123 Hz) and two ^103^Rh (^1^*J*_P–Rh_ = 114 Hz) nuclei, whereas the outer phosphines gave rise to a single, doublet-of-doublets resonance from coupling to ^103^Rh (^1^*J*_P–Rh_ = 178 Hz) and the ^31^P of the phosphide. If **4** could dissociate into putative (PPP)Rh monomers, they would be expected to undergo an oxidative addition reaction with aryl halides at Rh, by analogy with other (pincer)Rh^I^ fragments.^17,18^ However, thermolysis of **4** at 75° for 20 h in C_6_D_6_ in the presence of PhBr did not lead to any observable changes. This suggests that **4** does not dissociate into monomers easily.

The ^31^P{^1^H} NMR spectrum of **5** presented a phosphide resonance at δ 218.9 ppm and two distinct resonances at 38.1 and 34.0 ppm, indicating the inequivalence of the −P^i^Pr_2_ arms on the NMR time scale.

The ^1^H NMR spectrum of **6** contained broadened resonances indicative of 2-fold symmetry within the PPP ligand. In the ^31^P{^1^H} NMR spectrum, two overlapping resonances devoid of discernible fine structure were observed at ambient temperature around 44–46 ppm; one was much broader than the other. We examined the ^31^P{^1^H} NMR spectra of **6** in the range of temperatures from −75 to +95 °C in toluene-d_8_ under a CO atmosphere (to prevent decomposition at higher temperatures). The observations indicated that one of these resonances belongs to the two ^31^P nuclei of the central bridging phosphides, while the other represents the four ^31^P nuclei in the -PPr^i^_2_ arms. The latter clearly resolves into two multiplets at −55 °C and coalesces into a single resonance upon warming. The phosphide resonance largely retains its shape above −55 °C.

### XRD Structural Studies of **4**, **5**, and **6**

**Figure 3:**
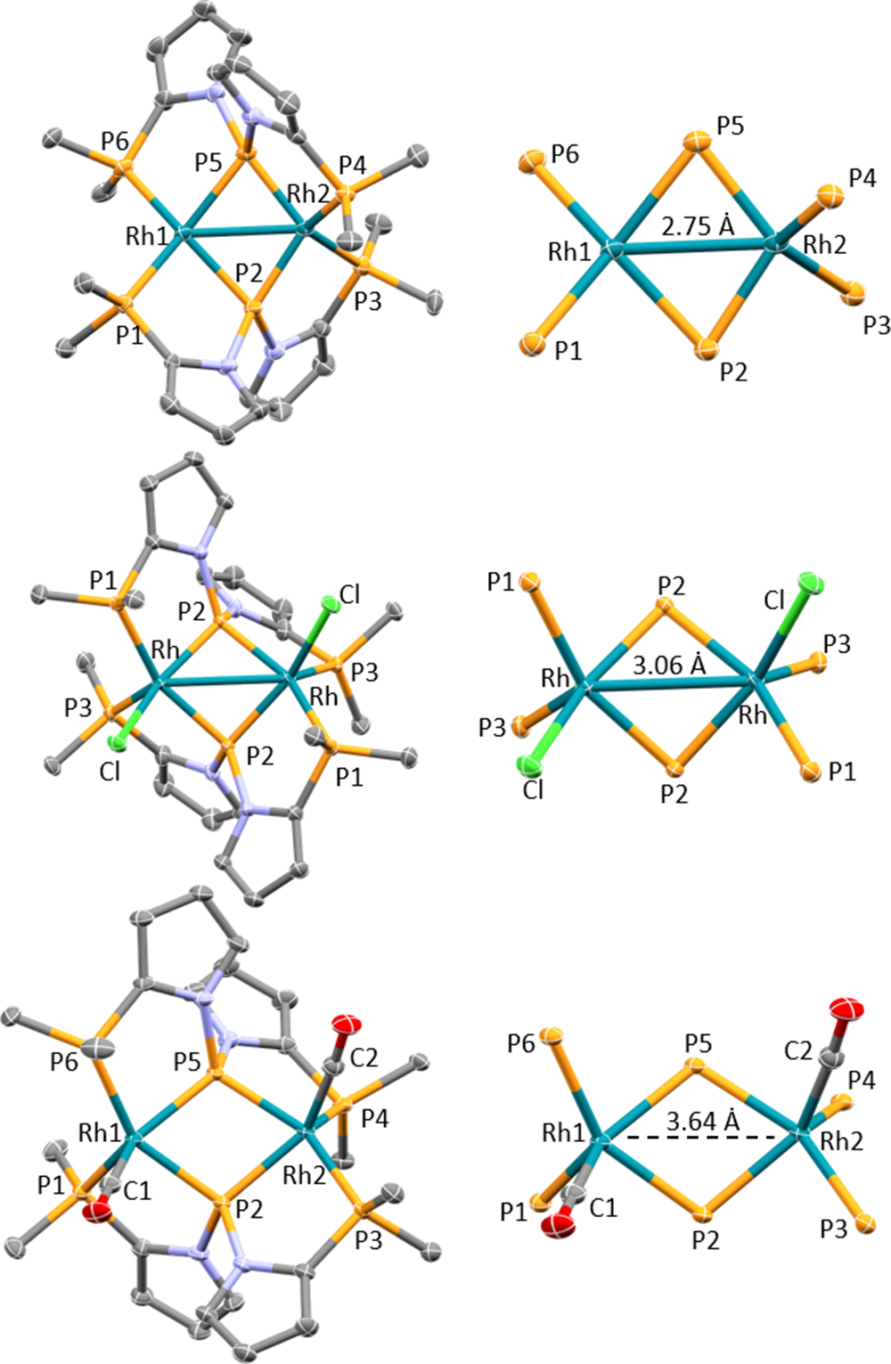
*ORTEP drawing of***4** (top), **5** (middle), and **6** (bottom). Hydrogen atoms and methyl groups on the isopropyl arms are omitted for clarity. Only one of the two independent molecules in the asymmetric unit of **4** is shown. Solvent of crystallization (pentane for **6**) is omitted. The drawings on the right show only the immediate coordination environment for the Rh_2_ units in each molecule. For 4, selected distances (Å): Rh1–Rh2: 2.751(1), Rh1–P1: 2.273(3), Rh1–P2: 2.308(2), Rh1–P5: 2.300(3), Rh1–P6: 2.288(3), Rh2–P2: 2.148(3), Rh2–P3: 2.305(2), Rh2–P4: 2.293(2), and Rh2–P5: 2.142(2). For 5, selected distances (Å): Rh–Rh: 3.0643(4), Rh–P1: 2.3634(8), Rh–P2: 2.1906(7), Rh1–P3: 2.3262(7), and Rh–Cl: 2.4561(7). For 6, selected distances (Å): Rh1–Rh2: 3.6406(6), Rh1–P1: 2.4156(9), Rh1–P2: 2.3021(9), Rh1–P5: 2.3352(8), Rh1–P6: 2.3239(9), Rh1–C1: 1.874(3), Rh2–P2: 2.3232(8), Rh2–P3: 2.3279(8), Rh2–P4: 2.402(1), Rh2–P5: 2.2950(8), and Rh2–C2: 1.876(3).

**Figure 4:**
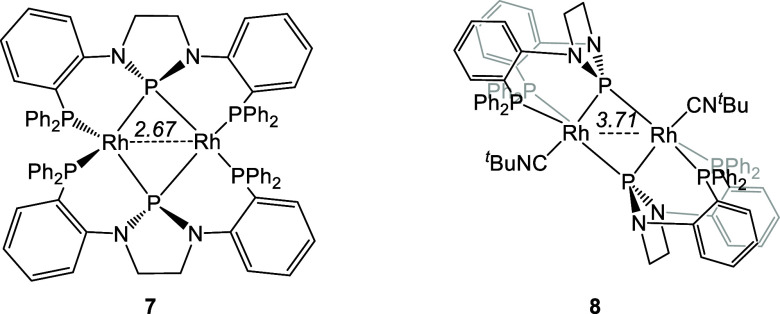
Bimetallic Rh complexes of a related PPP ligand were reported by the Thomas group. XRD-determined Rh–Rh separation (in Å) is given.

Solid-state structures of **4**–**6** were determined via XRD studies on suitable single crystals (Figure 3). The structures of **4**–**6** are all dimeric with the two phosphide ligands bridging the two Rh centers to form the Rh2P2 diamondoid at the core of each structure. The overall conformation of the PPP ligand in each of these three structures is roughly similar, and the two outer phosphines in each PPP ligand bind to different Rh centers. The three structures differ first in their disparate Rh–Rh distances, ranging from ca. 2.75 Å in **4** to ca. 3.06 Å in **5** and 3.64 Å in **6**. This disparity is also reflected in the different Rh–P–Rh angles for **4** (ca. 76°), **5** (ca. 87°), and **6** (ca. 104°). Second, the structures of **5** and **6** are C_2_-symmetric or nearly so. The two pairs of the intradiamondoid Rh–P distances differ only by <0.04 Å within each of these two molecules and in a staggered fashion, meaning that the complement of M–L distances about both Rh centers in **5** and (separately) in **6** is approximately the same. In contrast, in the structure of **4**, the two Rh centers possess meaningfully different coordination environments. One of the Rh centers connects to the bridging phosphidos with two short (2.13–2.14 Å) Rh–P bonds, while the other connects with two long (2.29–2.31 Å) Rh–P bonds. Ignoring the Rh–Rh contact, the coordination environment about the Rh1 center with the longer Rh–P(phosphido) bonds is somewhat closer to square-planar, while the other (Rh2) is closer to tetrahedral. This can be exemplified by the fact that the two largest P–Rh–P angles about the “square-planar” Rh1 are ca. 150°, but the two largest P–Rh–P angles about the “tetrahedral” Rh2 are only ca. 125–130°. A degenerate isomerization that interconverts the two different Rh positions (for example, by shifting the bridging phosphidos closer/farther from the Rh centers) would require relatively modest intramolecular motions. This is probably why **4** displays higher symmetry (all −PPr^i^_2_ arms equivalent) in the ^31^P{^1^H} NMR spectra at RT. Our observations here are quite similar to those made by the Thomas group on a related [(PPP)Rh]2 dimer complex **7** (Figure 4).^27^ DFT calculations performed by the Thomas group indicated that the symmetric Rh2P2 structure is only 10.5 kcal/mol higher than the asymmetric structure, also leading to symmetric time-averaged NMR spectra. The Thomas group also reported compound **8**, which can be viewed as analogous to **6**, but bearing isonitriles instead of CO. However, the connectivity in **8** is different: while the phosphide still bridges two different Rh centers, the -PR_2_ arms of each PPP ligand bind to the same Rh in **8**, but to different Rh centers in **6**.

The two types of -PPr^i^_2_ arms in the structures of **5** and **6** can be viewed as different because one type is approximately *trans* to one of the P_phosphido_ atoms, while the other one is approximately *cis* to both. The two largest P_arm_–Rh–P_phosphido_ angles at each Rh center in the structures of both **5** and **6** are ca. 100–110° and 150–160°. For **5**, three distinct ^31^P NMR resonances were observed at RT (i.e., two different P^i^Pr_2_ groups), indicating that the barrier for the exchange of the two P^i^Pr_2_ groups is higher than that in **4** or **6**.

**Figure 5:**
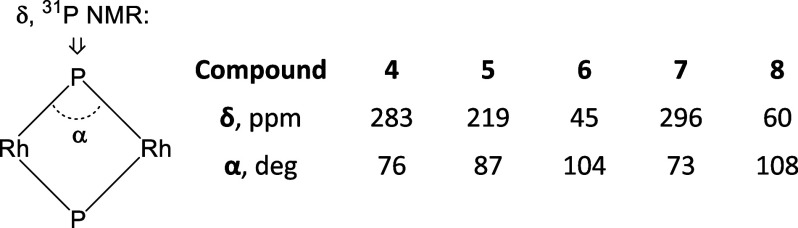
Apparent correlation between the Rh–P–Rh angle (from XRD) and the ^**31**^P NMR chemical shift for the bridging phosphide in compounds **3**–**7**.

We were surprised by the seemingly unusual ^31^P{^1^H} NMR chemical shift of the phosphide resonance in **6** (ca. 45 ppm), given that in the other two compounds (**4** and **5**), it is downfield of 200 ppm. We found some relevant insight into the studies of complexes with bridging phosphide ligands in the 1980s. For example, Meek and co-workers noted^28^ that the ^31^P NMR chemical shifts of a μ-PPh_2_ moiety could be as different as +205 ppm and −130 ppm in Pd and Pt complexes,^29−31^ ostensibly depending on the presence (more downfield) or absence (more upfield) of M–M bonding accompanying the μ-PPh_2_ bridge. It is not clear whether it is the M–M bonding that is truly responsible for the variation or simply the accompanying change in the M–P–M angle.^32^ Indeed, the chemical shift of the phosphide resonance trends upfield with the diminishing Rh–Rh bonding and the increasing Rh–P–Rh angle in the series **4**–**6**, and compounds **7** and **8** also conform to this trend (Figure 5).

### Theoretical Analysis

In order to better understand the observed structural features, we utilized DFT calculations at the M06/LANL2DZ/6-31G(d) level of theory^33^ within the Gaussian suite^34^ to obtain the optimized structures of **4**–**6**. Overall, the DFT-derived structures reproduced the key features of the X-ray structures reasonably well. The dissymmetry of **4** was reproduced as were the overall conformations of the PPP ligand about the Rh centers. The calculated Rh–Rh distances were somewhat longer than the XRD-derived distances (**4**: 2.903 Å, **5**: 3.093 Å, and **6**: 3.668 Å), but the relative ordering and the magnitude of the differences among the three compounds were reproduced. Rh–Rh Wiberg bond indices (WBI)^35^ were calculated to be 0.234, 0.256, and 0.065, whereas the Mayer^36^ bond indices were calculated to be 0.398, 0.297, and 0.055 for **4**–**6**, respectively.

The bonding situation in **6** is easiest to rationalize. Each Rh center has five donors about it, and thus each can be described as a five-coordinate d^8^ structure with the expected geometry intermediate between trigonal bipyramidal and square pyramidal (τ = 0.15^37^ in the XRD structure). The Rh–Rh distance is too long to contemplate bonding, as should be expected for a dimer with two saturated, 18-electron metal sites.

Compound **5** is a Rh^II^–Rh^II^ dimer. It might be expected that an octahedral environment was adopted about each Rh. However, because of all the chelate constraints, the deviations from orthoaxial geometry are severe, and the Rh–Rh distance of ca. 3.06 Å (XRD) in **5** is much longer than the Rh^II^–Rh^II^ distance in a carboxylate-bridged dimer such as Rh_2_(OAc)_4_(H_2_O)_2_ (2.3855(5) Å),^38^ or the typical unsupported Rh^II^–Rh^II^ bonds on the order of 2.7 Å.^39^ The long Rh–Rh distance in **5** and the modest WBI value suggest that the Rh–Rh interaction is probably not a simple two-center, two-electron Rh–Rh σ-bond. The Tantillo group has calculated the WBI value for the Rh–Rh bond in Rh_2_(OAc)_4_ to be 0.89,^40^ consistent with partial Rh–Rh bonding in **4** and **5**.

The structure of **4** is less straightforward to understand. Structural preferences of (pseudo)halide bridged dimers of the general formula L_2_M(μ-X)_2_ML_2_ were studied by Summerville and Hoffman in 1976.^41^ They specifically looked at d^8^–d^8^ dimers (including Rh^I^) in which the two local metal coordination environments were either tetrahedral or square-planar. One of Hoffman’s conclusions was that the nature of the bridging ligands is “determinative” and that a ligand such as chloride, possessing extra lone pairs of electrons, favors the bis(square-planar) motif to a greater degree than a phosphido (PH_2_) bridging ligand. They noted that π–π interactions of the metal’s d-orbitals with either the lone pair of the bridging chloride or the σ* of the P–H bond play a role in this preference. The presence of the more polarized P–N bonds in P2Rh2 (as well as in the Thomas compound **7**) and thus lower-energy and more P-based σ* orbitals should enhance this effect. In addition, Summerville and Hoffman posited that a π-bond exists between the two metals in the dimers, even at relatively large separations, whereas the presence of a σ-bond may be ambiguous.

**Figure 6:**
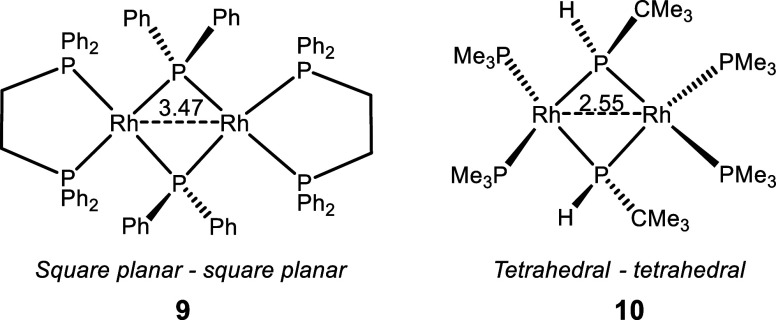
Representation of the structures of **9** and **10**; XRD-determined Rh–Rh separation (in Å) is given.

The known bis(square-planar) dimers tend to have long Rh–Rh separations, while the bis(tetrahedral) dimers possess shorter Rh–Rh contacts. Compounds **9**(27) and **10**(42) are two literature examples of this dichotomy with bridging phosphide ligands (Figure 6); like in **4**, the two Rh centers are supported by four phosphines and two bridging phosphidos. It is reasonable to suppose that the steric pressure of the substituents on the P atoms, as well as any chelate constraint present, should influence the preference for square-planar vs tetrahedral. It is likely that the smaller bite angle of the dppe ligand in **9** favors the square-planar geometry, whereas the steric pressure of the large *t*-Bu substituent in **10** may help bias the system toward tetrahedral.

**Figure 7:**
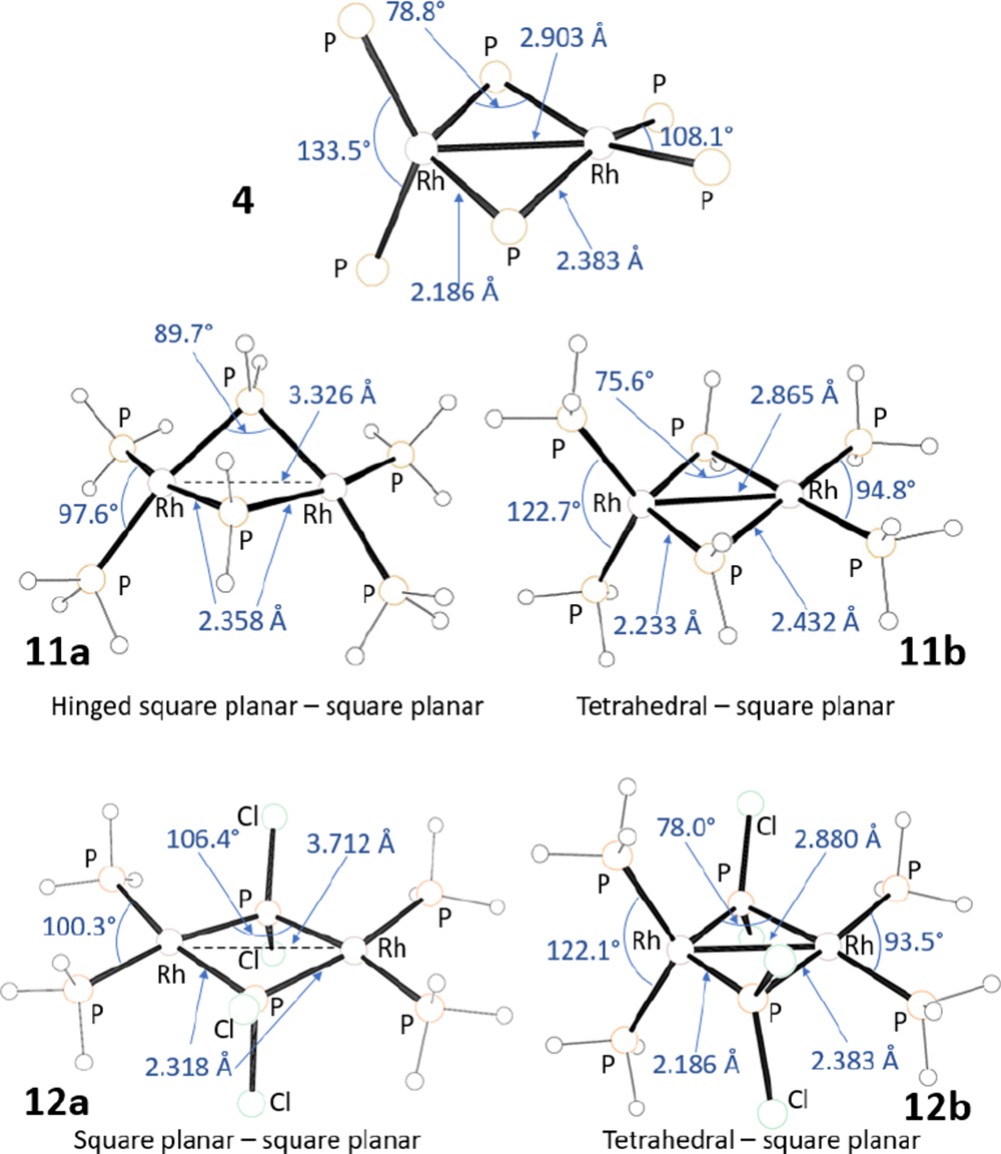
Representations of the DFT-calculated structures of **4** (only the Rh and P atoms are shown), **11a**, **11b**, **12a**, and **12b** (H atoms and two of the Cl atoms in **12b** are shown unlabeled), with selected DFT-derived metrics displayed.

Curious about the possible behavior in the absence of chelate constraints or significant steric concerns, we minimized the structures of (H_3_P)_2_Rh(μ-PH_2_)_2_Rh(PH_3_)_2_ (**11**, Figure 7) and (H_3_P)_2_Rh(μ-PCl_2_)_2_Rh(PH_3_)_2_ (**12**, Figure 7). We attempted to find minima corresponding to the bis(square-planar) and bis(tetrahedral) dimers of D_2h_ symmetry for both **11** and **12**. However, the D_2h_ symmetry did not appear to correspond to minima. The bis(square-planar) nature of the dimers was preserved upon optimization, but it resulted in the “hinged” geometry about the bridging P atoms, which was more pronounced in **11a** than in **12a**. Summerville and Hoffman pointed out the shallow potential energy surface for the mild hinging distortion.^39^ These dimers are symmetric and possess long (**11a**: 3.326 Å and **11b**: 3.712 Å) Rh–Rh separations and small WBI values of 0.131 and 0.077, respectively. The other minima **11b** and **12b** found were dissymmetric hybrid tetrahedral/square-planar dimer with shorter Rh–Rh distances (**11b**: 2.865 Å and **12b**: 2.880 Å). These distances (Figure 7) and WBI values (**11b**: 0.287 and **12b**: 0.252) are similar to the calculated Rh–Rh distance (2.903 Å, Figure 7) and the WBI value in **4** (0.234). Compared to **4**, the two different coordination geometries about the Rh atoms of the calculated dimers **11b** and **12b** are more rigorously idealized, but on the whole, **4** possesses a closely related structure.

For **11**, the square-planar/square-planar isomer **11a** is favored by 11.1 kcal/mol in energy over the tetrahedral/square-planar isomer **11b**. However, for **12**, the corresponding isomer **12a** is calculated to be preferred only by 0.4 kcal/mol in free energy. These calculations indicate that the dissymmetric tetrahedral/square-planar is favored to a greater degree with the more electronegative substituents on the bridging P (Cl in **12** vs H in **11**). An N-pyrrolyl is electronically similar to Cl; thus, there must also be some intrinsic preference for the observed structure of **4** besides the constraints of the chelating ligands.

## Conclusions

In summary, we successfully installed a new phosphido/bis(phosphine) PPP ligand in the coordination sphere of Rh. Interestingly, this ligand, conceived as a pincer, prefers to form bimetallic complexes with Rh in which the two outer -P^i^Pr_2_ arms of the “pincer” bind to different Rh centers. The geometry of the Rh^I^–Rh^I^ [(PPP)Rh]_2_ dimer (compound **4**) is rather unusual in that it contains two different Rh centers: one in a distorted tetrahedral environment and one in a distorted square-planar environment. DFT investigations and other evidence in the literature suggest that this type of geometry is indeed one of the possible minima for the [(μ-phosphido)Rh(phosphine)_2_]_2_ systems, especially with electron-poor phosphidos, although the factors controlling the exact geometry preferences of such dimers are subtle.

## Supporting Information Available

Experimental details and NMR spectra (PDF)
Crystallographic information found in the Cambridge Structural Database under entries CCDC 1911259-1911262 (ZIP)
