# Supplementary material for: Dimeric Rh Complexes Supported by a Bridging Phosphido/Bis(Phosphine) PPP Ligand
Source: Organometallics. 2024 Apr 22;43(9):947–53. doi: 10.1021/acs.organomet.3c00492 (PMC11094786; doi:10.1021/acs.organomet.3c00492)
Supplement: Supplementary file 1 — om3c00492_si_001.pdf [file om3c00492_si_001.pdf]

Supporting Information

Dimeric Rh Complexes Supported by a Bridging Phosphido/Bis(Phosphine) PPP Ligand

Mario N. Cosio,[†] Samuel R. Lee,[†] Qingheng Lai,[†] Nattamai Bhuvanesh,[†] Jia Zhou,[‖] and Oleg V. Ozerov[*,†]

[†] *Department of Chemistry, Texas A&M University, College Station, Texas 77842, United States*

[‖] *State Key Laboratory of Urban Water Resources and Environment, School of Science, Harbin Institute of Technology, Shenzhen 518055, China*

ozerov@chem.tamu.edu and jiazhou@hit.edu.cn

I. GENERAL CONSIDERATIONS 3

II. SYNTHESIS AND CHARACTERIZATION DETAILS 4

III. X-RAY STRUCTURAL DETERMINATION DETAILS 26

IV. COMPUTATIONAL METHODS 32

V. SI REFERENCES 33

I. General Considerations

Unless specified otherwise, all manipulations were performed under an Ar atmosphere using standard Schlenk line or glovebox techniques. Toluene, tetrahydrofuran (THF), diethyl ether, isooctane, and pentane were dried and deoxygenated (by sparging with argon) using an Innovative Technologies MD-5 solvent purification system and stored over molecular sieves in an Ar-filled glovebox. Triethylamine (NEt_3), C_7D_8, and C_6D_6 were dried over NaK/Ph_2CO/18-crown-6, while acetonitrile (CH_3CN), $CDCl_3$, and fluorobenzene (PhF) were dried over CaH_2. The solvents were distilled or vacuum transferred and stored over molecular sieves in an Ar-filled glovebox. Magnesium powder was activated by stirring in diethyl ether with iodine. Metal and ligand precursors $[Rh(COD)Cl]_2$[1] and **1**[2] were synthesized according to literature precedent. All other chemicals were used as received from commercial vendors. NMR spectra were recorded on Bruker Avance 400 (^{1}H NMR, 399.535 MHz; ^{13}C NMR, 100.465 MHz), Avance Neo 400 (^{1}H NMR, 400.200 MHz; ^{13}C NMR, 100.630 MHz; ^{31}P NMR, 161.95 MHz) Avance 500 (^{1}H NMR, 500.130 MHz; ^{13}C NMR, 125.758 MHz), Inova 500 (^{1}H NMR, 499.431 MHz; ^{13}C NMR, 125.595 MHz; ^{31}P NMR, 202.187 MHz), and Avance Neo 500 (^{1}H NMR, 500.13 MHz; ^{13}C NMR, 125.77 MHz; ^{31}P NMR, 202.45 MHz) spectrometer. Chemical shifts are reported in δ (ppm). For ^{1}H and ^{13}C NMR spectra, the residual solvent peak was used as an internal reference (^{1}H NMR: δ 7.16 for C_6D_6, 7.26 for $CDCl_3$; ^{13}C NMR: δ 128.06 for C_6D_6, 77.16 for $CDCl_3$). ^{31}P NMR spectra were externally referenced to an 85% phosphoric acid solution *δ* 0.00. Elemental analyses were performed by CALI Labs, Inc. (Parsippany, NJ).

II. Synthesis and Characterization Details

Synthesis of 2. 2-diisopropylphosphinopyrrole (**1**; 0.586 g, 3.20 mmol) was added to a 50 mL Schlenk flask and dissolved in ca. 5 mL of THF. An excess of NEt_3 was added (2.75 mL, 19.20 mmol) followed by PCl_3 (0.130 mL, 1.52 mmol). Adding PCl_3 before NEt_3 results in lower product yield. Upon addition of PCl_3, a white precipitate (presumed to be Et_3NHCl) was formed. The mixture was stirred for 1 h at room temperature then the volatiles were removed under vacuum. The crude residue was extracted with isooctane, filtered, and then the volatiles were removed again under vacuum to afford a white oil. The oil was recrystallized by dissolving it in acetonitrile and placing it in a -38 °C freezer to afford **2** as a white solid precipitate. (0.31 g, 47% yield, >95% purity by NMR). ^{1}H NMR (500 MHz, C_6D_6): δ 7.11 (s, pyrrole ring, 2H), 6.40 (s, pyrrole ring, 2H), 6.35 (s, pyrrole ring, 2H), 1.84-1.90 (m, C*H*Me_2, 4H), 0.95-1.06 (m, CH*Me_2*, 18H), 0,87-0.90 (m, 6H, CH*Me_2*). ^{13}C{^{1}H} NMR (100 MHz, C_6D_6): δ 132.1 (dd, $J_{C\text{-}P}$ = 9 Hz, $J_{C\text{-}P}$ = 22 Hz), 126.4 (dvt, $J_{C\text{-}P}$ = 2 Hz, $J_{C\text{-}P}$ = 6 Hz), 120.34 (m), 114.1 (m), 25.2 (dd, $J_{C\text{-}P}$ = 4 Hz, $J_{C\text{-}P}$ = 10 Hz), 24.2 (dd, $J_{C\text{-}P}$ = 6 Hz, $J_{C\text{-}P}$ = 8 Hz), 20.3 (d, $J_{C\text{-}P}$ = 18 Hz), 20.2 (d, $J_{C\text{-}P}$ = 21 Hz), 19.7 (d, $J_{C\text{-}P}$ = 11 Hz), 18.78 (m). ^{31}P{^{1}H} NMR (202 MHz, C_6D_6): δ 102.6 (t, $J_{P\text{-}P}$ = 205 Hz, P*P*P), -20.6 (d, $J_{P\text{-}P}$ = 206 Hz, P^iPr_2).

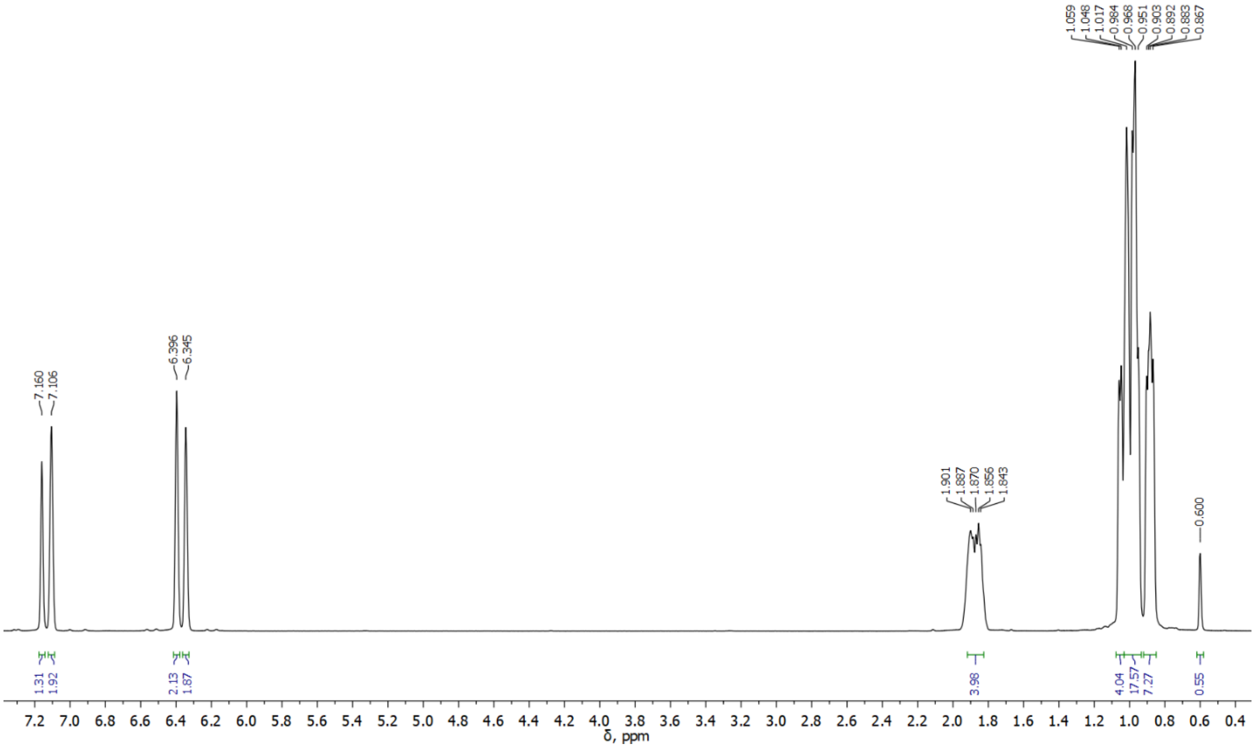

Figure S1. ^{1}H NMR (500 MHz, C_6D_6) of **2**.

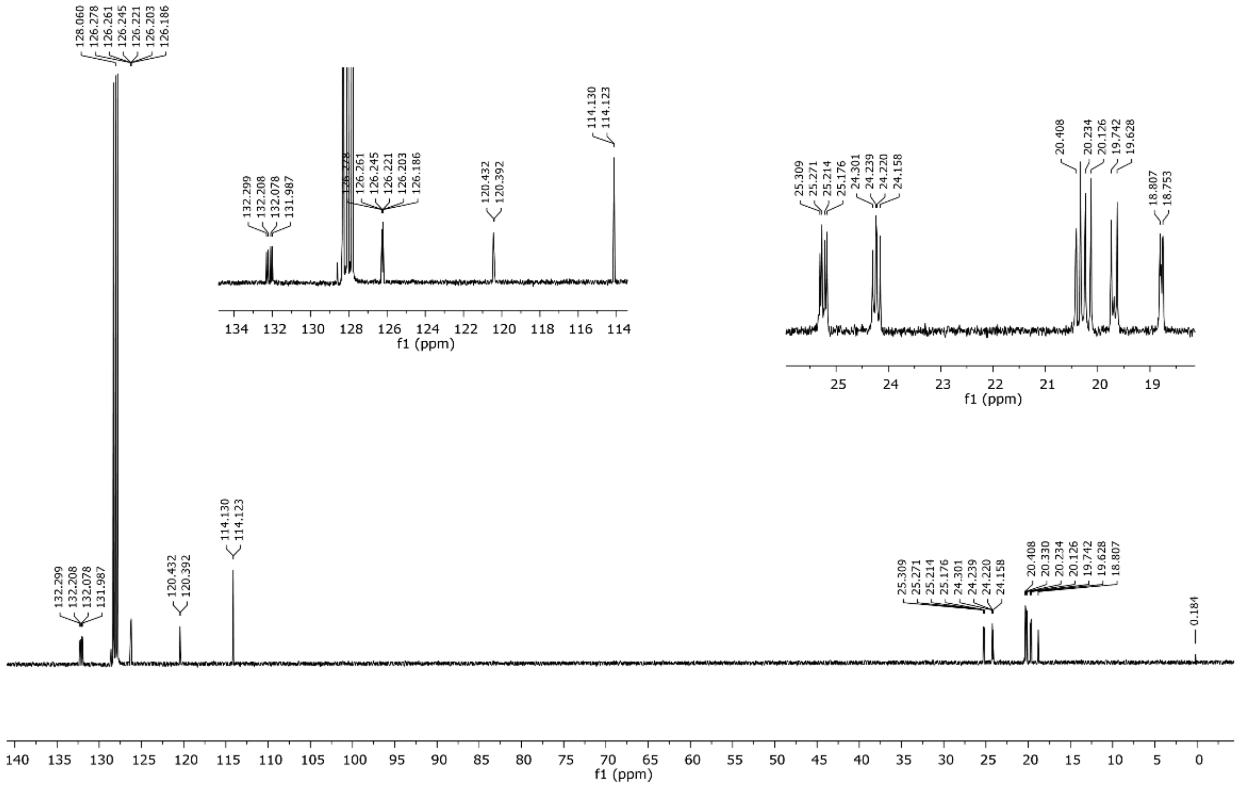

Figure S2. ^{13}C{^{1}H} NMR spectrum (100 MHz, C_6D_6) of **2**.

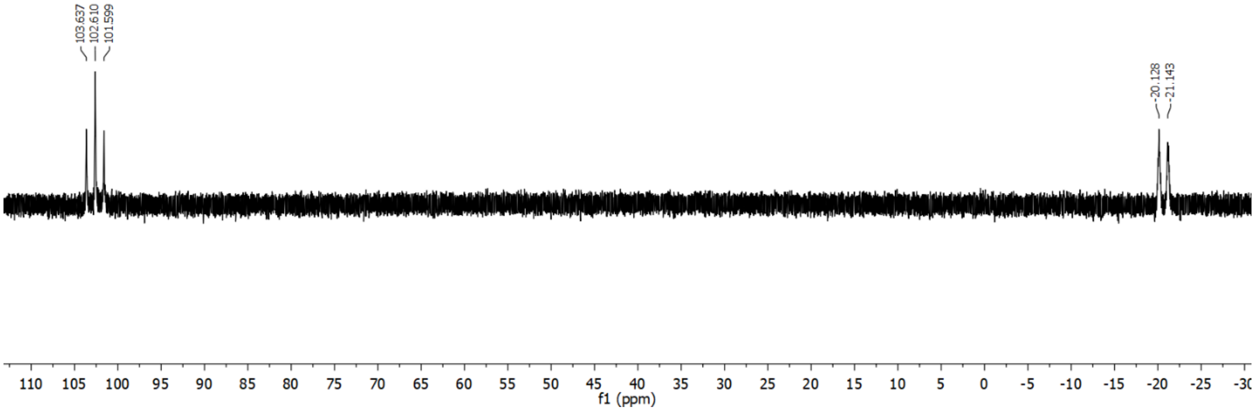

Figure S3. $^{31}P\{^1H\}$ NMR spectrum (202 MHz, C_6D_6) of **2**.

Synthesis of 3a. In a 50 mL Schlenk flask, **2** (1.3 g, 3 mmol) and $[Rh(COD)Cl]_2$ (740 mg, 1.5 mmol) were combined and dissolved in minimal toluene, stirred for 15 min, and the volatiles were removed under vacuum. The crude solid was stirred vigorously in isooctane and the solids were collected by filtration to afford 1.4 g of **3a** as a yellow powder (90% yield). Single crystals were grown by cooling a saturated solution of **3a** in PhF in a -38 °C freezer overnight. ^1H NMR (400 MHz, $CDCl_3$): δ 7.46 (br s, pyrrole ring, 2H) , 6.79 (t, *J* = 3.1 Hz, pyrrole ring, 2H), 6.49 (m, pyrrole ring, 2H), 2.85 (sept, *J* = 6.9 Hz, C*H*Me_2, 2H), 2.47 (m, C*H*Me_2, 2H), 1.28 – 1.39 (m, CHMe_2, 12H), 1.23 (dvt, $J_{H\text{-}P} \approx J_{H\text{-}H}$ = 8.2 Hz, CHMe_2, 6H), 1.09 (dvt, $J_{H\text{-}P} \approx J_{H\text{-}H}$ = 7.8 Hz, CHMe_2, 6H). $^{13}C\{^1H\}$ NMR (100 MHz, $CDCl_3$): δ 134.1 (ddt, $J_{C\text{-}Rh}$ = 2.1 Hz, $J_{C\text{-}P}$ = 24.1 Hz, $J_{C\text{-}P}$ = 44.9 Hz), 123.3, 120.8, 115.8 (d, $J_{C\text{-}P}$ = 18.8 Hz), 27.9 (vt, $J_{C\text{-}P}$ = 12.0 Hz), 24.8 (vt, $J_{C\text{-}P}$ = 12.8 Hz)

19.6, 19.4, 18.9 (vt, J_{C-P} = 4.1 Hz), 17.8. $^{31}P\{^{1}H\}$ NMR (202 MHz, $CDCl_3$): δ 105.4 (dt, J_{P-P} = 44 Hz, J_{Rh-P} = 285 Hz), 38.5 (dd, J_{P-P} = 44 Hz, J_{Rh-P} = 124 Hz). Elem. Anal. Found (Calculated) for $C_{20}H_{34}Cl_2N_2P_3Rh$: C, 42.48 (42.20); H, 6.08 (6.02); N 4.85 (4.92).

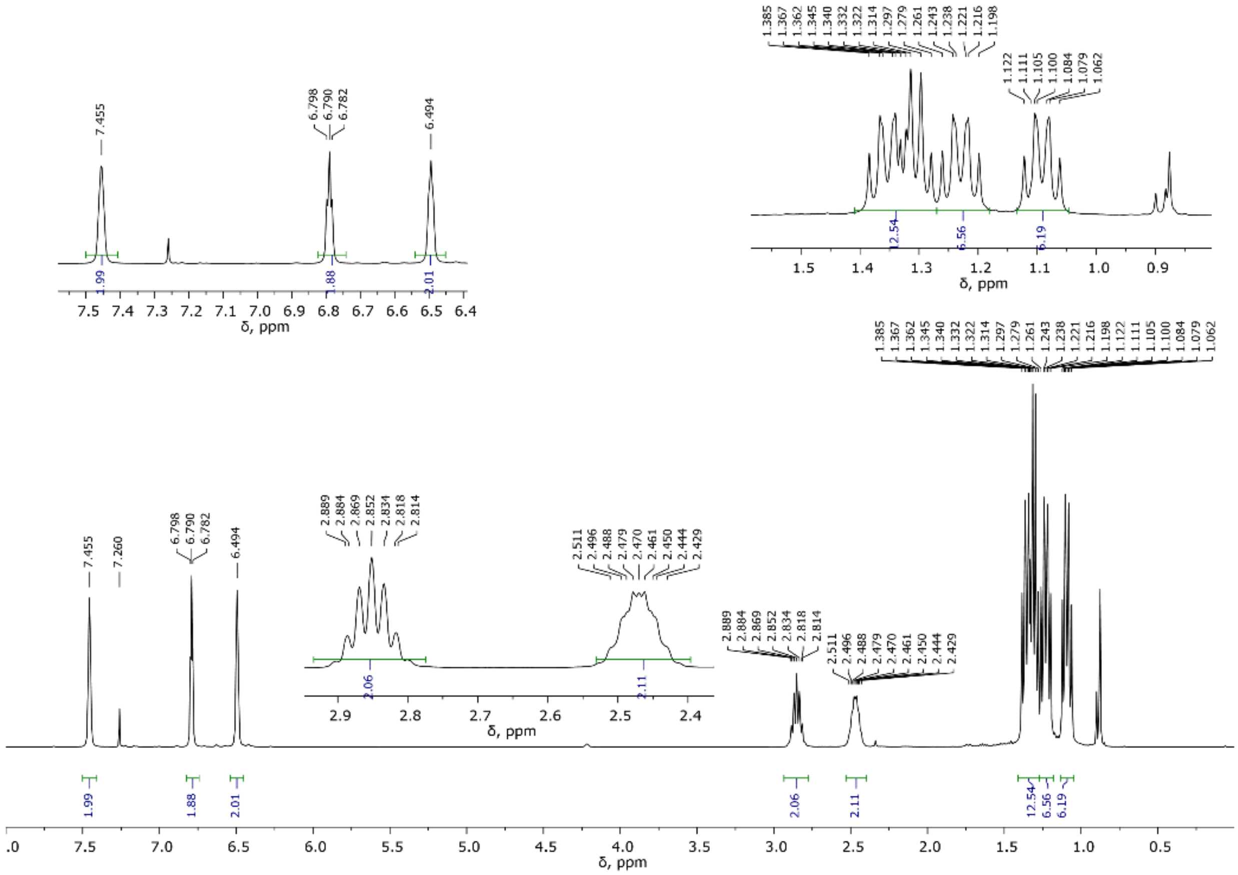

Figure S4. ^{1}H NMR spectrum (400 MHz, $CDCl_3$) of **3a**. Residual isooctane visible at 0.90 and 0.88 ppm.

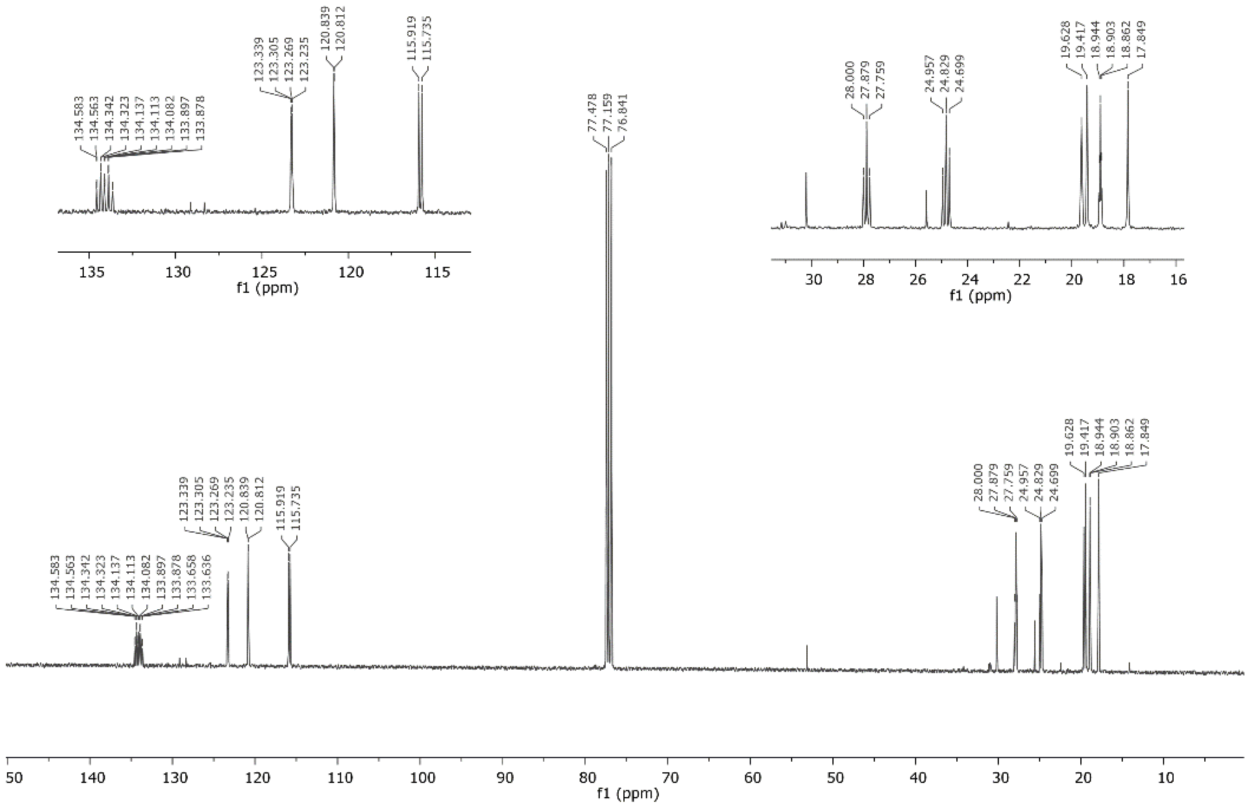

Figure S5. $^{13}C\{^{1}H\}$ spectrum (100 MHz, $CDCl_3$) of **3a**. Residual isooctane resonances visible at 53.1, 30.2, and 25.6 ppm.

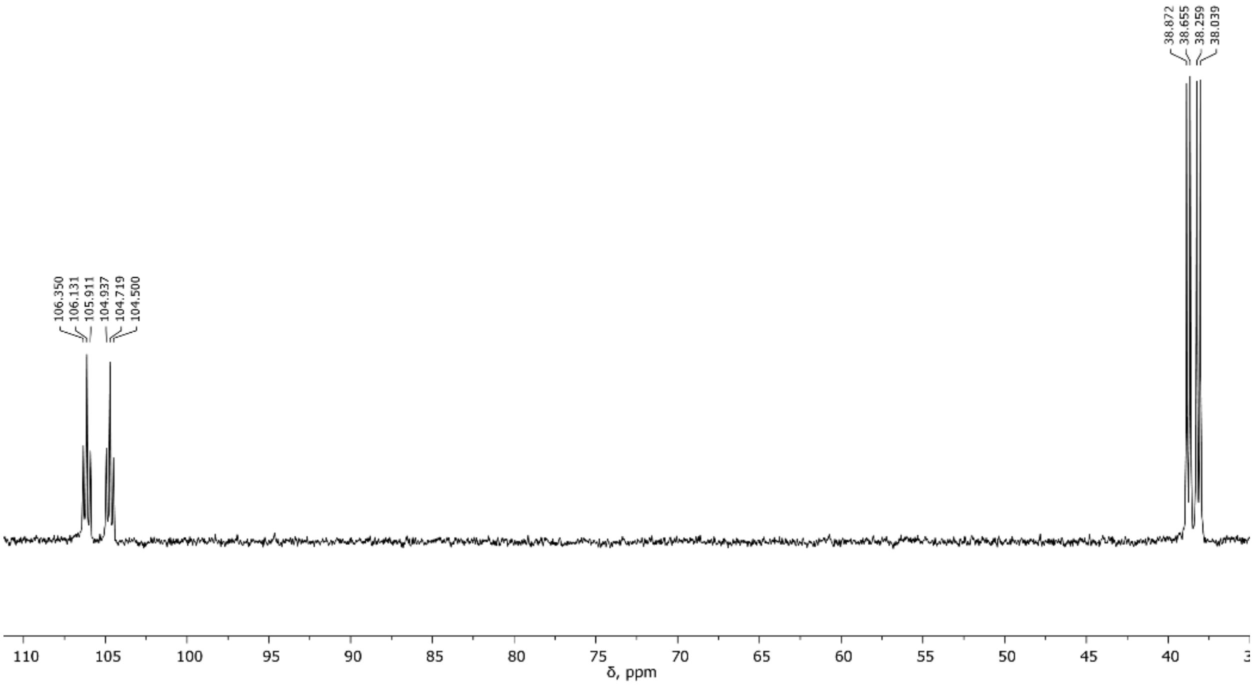

Figure S6. $^{31}P\{^1H\}$ NMR spectrum (202 MHz, $CDCl_3$) of **3a**.

***In situ* generation of 3b.** To a J. Young NMR tube was added 26 mg of **2** (0.06 mmol) and 15 mg $[Rh(COD)Cl]_2$ (0.03 mmol) dissolved in 0.6 mL C_6D_6 and stirred for 5 minutes before addition of Me_3SiBr (8 μL, 0.06 mmol). After 10 minutes, **3b** was observed as the sole product by $^{31}P\{^1H\}$ NMR. $^{31}P\{^1H\}$ NMR (202 MHz, C_6D_6) δ 104.9 (dt, $J_{P\text{-}P}$ = 42 Hz, $J_{Rh\text{-}P}$ = 289 Hz), 38.6 (dd, $J_{P\text{-}P}$ = 42 Hz, $J_{Rh\text{-}P}$ = 123 Hz).

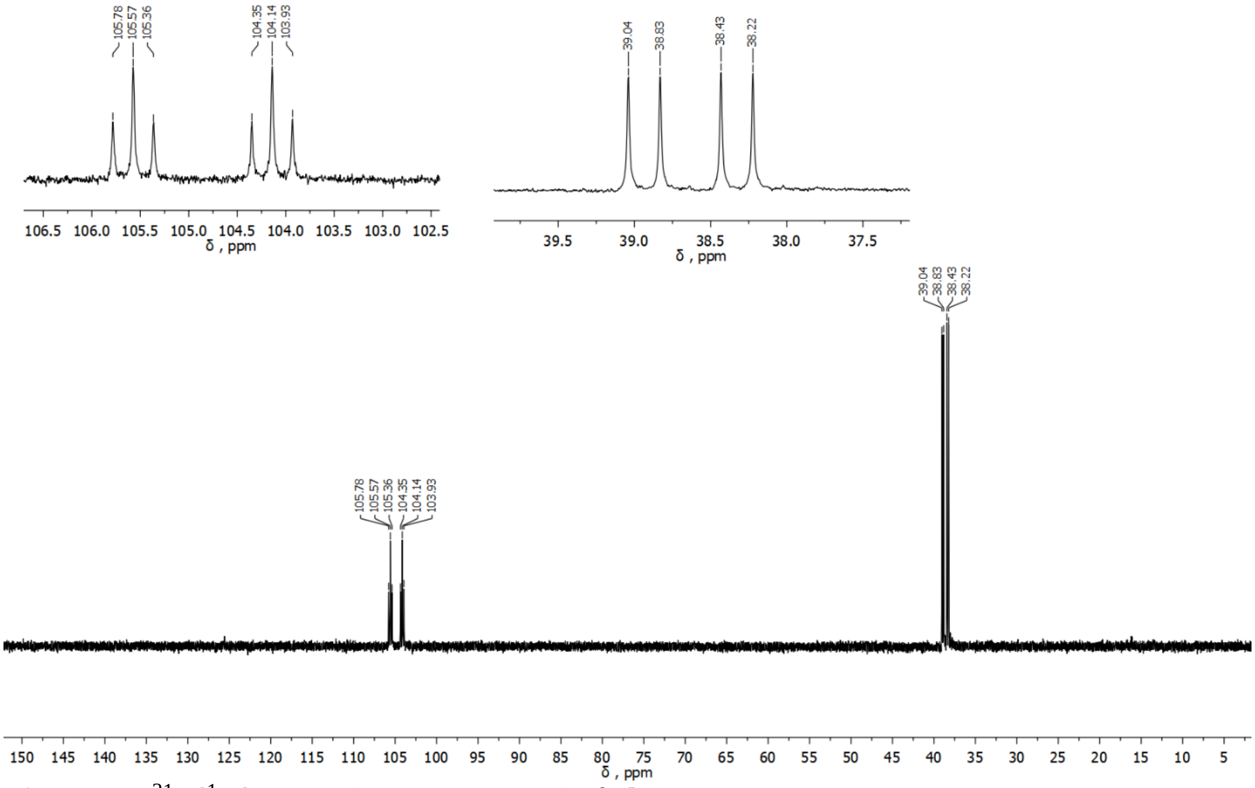

Figure S7. $^{31}P\{^{1}H\}$ NMR (202 MHz, C_6D_6) of **3b**.

***In situ* generation of 3a-d.** To a 20 mL scintillation vial charged with stirbar was added **2** (26 mg, 0.06 mmol) and dissolved in 0.7 mL C_6D_6 followed by addition of Me_3SiBr (8 μL, 0.06 mmol) and the solution was stirred for 5 minutes. To this solution was added $[Rh(COD)Cl]_2$ (15 mg, 0.03 mmol) with stirring for 15 minutes. The resulting mixture was characterized by means of $^{31}P\{^{1}H\}$ NMR: **3a**, **3b**, **3c**, and **3d** in ca. 1:2:1:2 ratio respectively, based on integrations of the $^{31}P\{^{1}H\}$ NMR peaks corresponding to the central phosphorus. $^{31}P\{^{1}H\}$ NMR data (202 MHz, C_6D_6) for **3a**: δ 105.2 (dt, $J_{P\text{-}P}$ = 44 Hz, $J_{Rh\text{-}P}$ = 285 Hz), 38.2 (dd, $J_{P\text{-}P}$ = 44 Hz, $J_{Rh\text{-}P}$ = 124 Hz); for **3b**: δ 104.9 (dt, $J_{P\text{-}P}$ = 42 Hz, $J_{Rh\text{-}P}$ = 289 Hz), 38.6 (dd, $J_{P\text{-}P}$ = 42 Hz, $J_{Rh\text{-}P}$ = 123 Hz); for **3c**: δ 84.8 (dt, $J_{P\text{-}P}$ = 44 Hz, $J_{Rh\text{-}P}$ = 285 Hz), 37.7 (dd, $J_{P\text{-}P}$ = 44 Hz, $J_{Rh\text{-}P}$ = 123 Hz); **3d**: δ 84.4 (dt, $J_{P\text{-}P}$ = 40 Hz, $J_{Rh\text{-}P}$ = 291 Hz), 38.1 (dd, $J_{P\text{-}P}$ = 40 Hz, $J_{Rh\text{-}P}$ = 122 Hz).

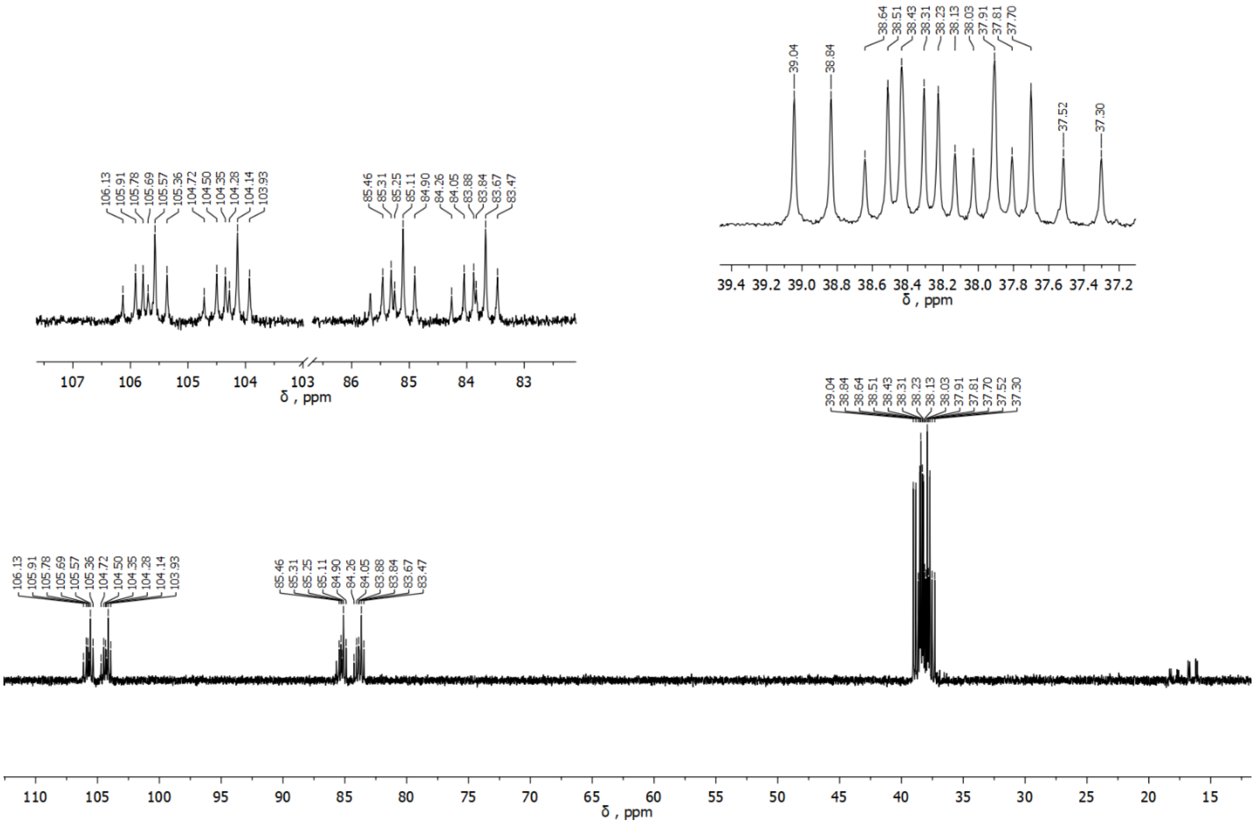

Figure S8. $^{31}P\{^1H\}$ NMR spectrum (202 MHz, C_6D_6) of the mixture containing **3a**, **3b**, **3c**, and **3d**.

Synthesis of 4. In a 20 mL scintillation vial equipped with stir bar, **3a** (508 mg, 0.892 mmol) was dissolved in 10 mL of THF. To the resultant solution, Mg (37 mg, 1.52 mmol) shavings were added. The mixture was stirred for 6 h, during which time the color changed from dark-orange to black. Tracking the reaction by $^{31}P\{^1H\}$ NMR shows the product was converted cleanly from **3a** to **4**. The solution was filtered to remove excess Mg and then pumped down to afford a black powder. The crude product was extracted with pentane and filtered through a celite plug. Volatiles were then removed by vacuum. The solid was washed with acetonitrile, triturated with isooctane, and dried under reduced pressure to afford a dark brown solid (0.32 g, 74% yield). Single crystals suitable for XRD were grown by dissolving the solid in pentane, filtering the solution into a small

vial, placing the vial into a larger vial with 3 mL hexamethyldisiloxane and then capping the larger vial and placing it in a -38 °C freezer for 3 days. The resultant crystals were black/brown and shined red under a polarized microscope. ^{1}H NMR (400 MHz, C_6D_6): δ 7.00 (s, 4H, pyrrole ring), 6.61 (t, *J* = 3.0 Hz, 4H, pyrrole ring), 6.28 (d, *J* = 3 Hz, 4H, pyrrole ring), 2.31 (sep, *J* = 6.6 Hz, 4H, C*H*Me$_2$), 1.87 – 2.03 (br m, 4H, C*H*Me$_2$), 1.28 (dd, *J* = 16.7, 6.9 Hz, 12H, CH*Me$_2$*), 1.21 (dd, *J* = 11.5, 6.7Hz, 12H, CH*Me$_2$*), 0.93 (dd, *J* = 16.3, 6.9 Hz, 12H, CH*Me$_2$*), 0.46 (dd, *J* = 16.1, 6.8 Hz, 12H, CH*Me$_2$*). ^{13}C{^{1}H} (100 MHz, C_6D_6): δ 134.4 (dvt, $J_{C\text{-}P}$ = 51.7 Hz, $J_{C\text{-}P}$ = 18 Hz) , 123.6, 116.6, 113.9, 29.8 (d, $J_{C\text{-}P}$ = 15 Hz), 26.9 (d, $J_{C\text{-}P}$ = 26.1 Hz), 21.0, 20.1, 19.2, 17.7. ^{31}P{^{1}H} (202 MHz, C_6D_6): δ 282.9 (tt, $J_{P\text{-}P}$ = 123, $J_{Rh\text{-}P}$ = 114 Hz, P*P*P), 57.2 dd, $J_{Rh\text{-}P}$ = 178 Hz, $J_{P\text{-}P}$ = 123 Hz, *P*^{i}Pr$_2$). Elem. Anal. Found (Calculated) for $C_{40}H_{68}N_4P_6Rh_2$ C, 48.05 (48.20); H, 6.86 (6.88); N, 5.49 (5.62).

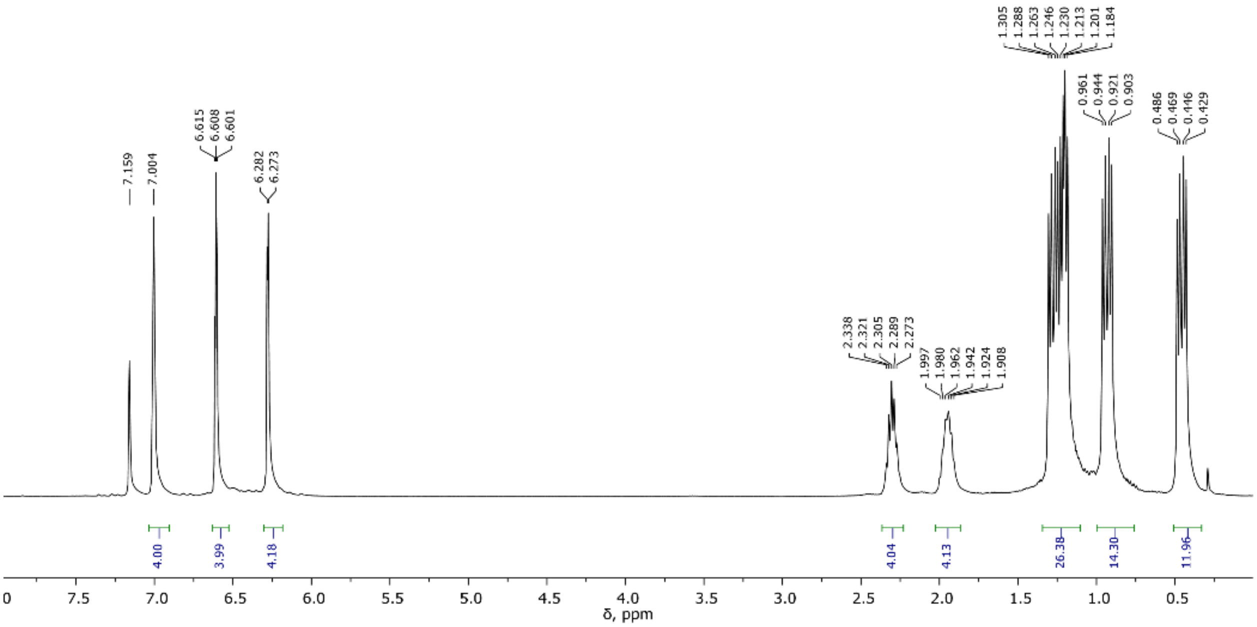

Figure S9. ^{1}H NMR spectrum (400 MHz, C_6D_6) of **4**.

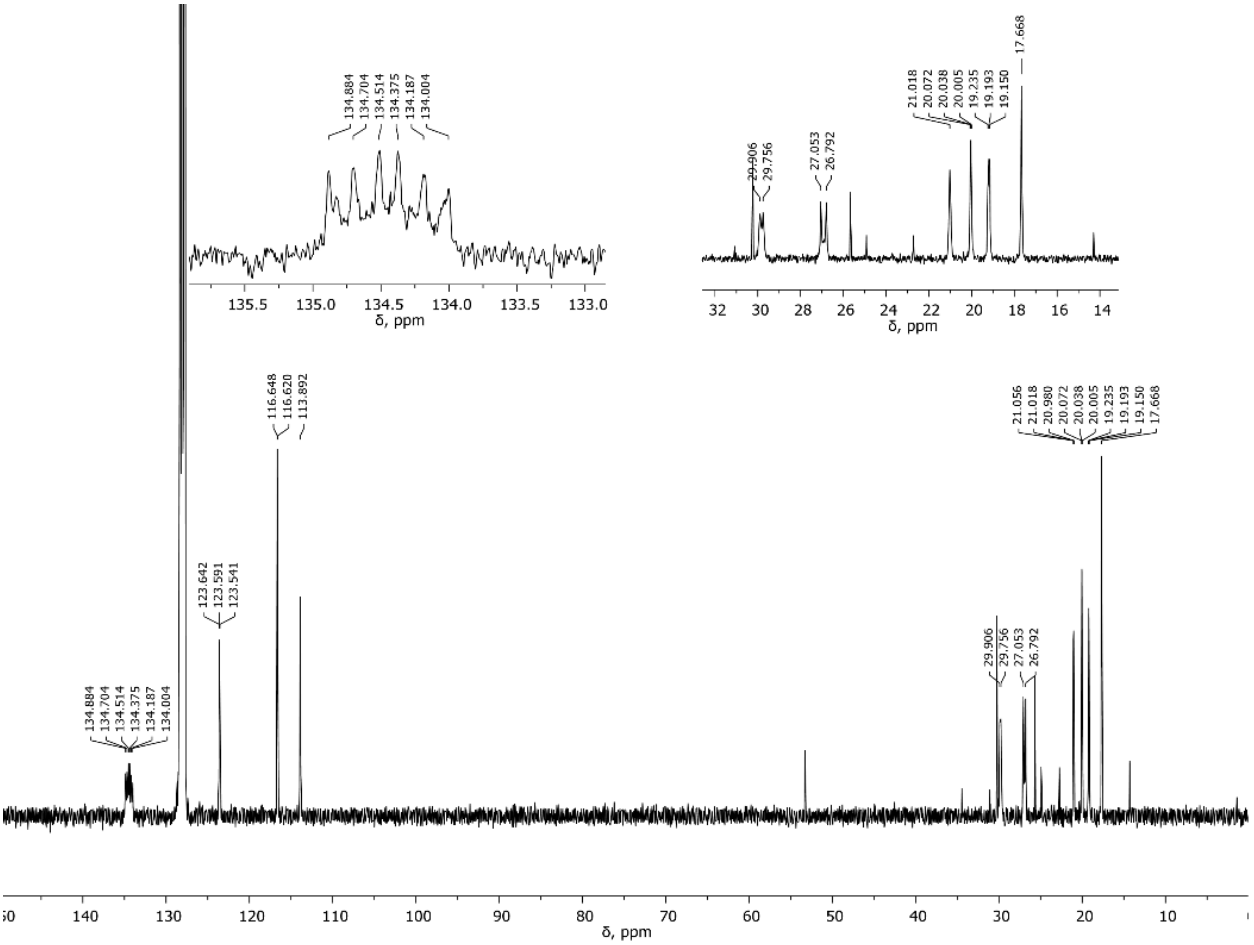

Figure S10. ${}^{13}C\{{}^{1}H\}$ NMR spectrum (100 MHz, C_6D_6) of **4**. Residual isooctane is visible at 53.3, 31.1, 30.2, 25.7, and 24.9 ppm. Residual pentane resonances at 34.4, 22.7, and 14.3 ppm.

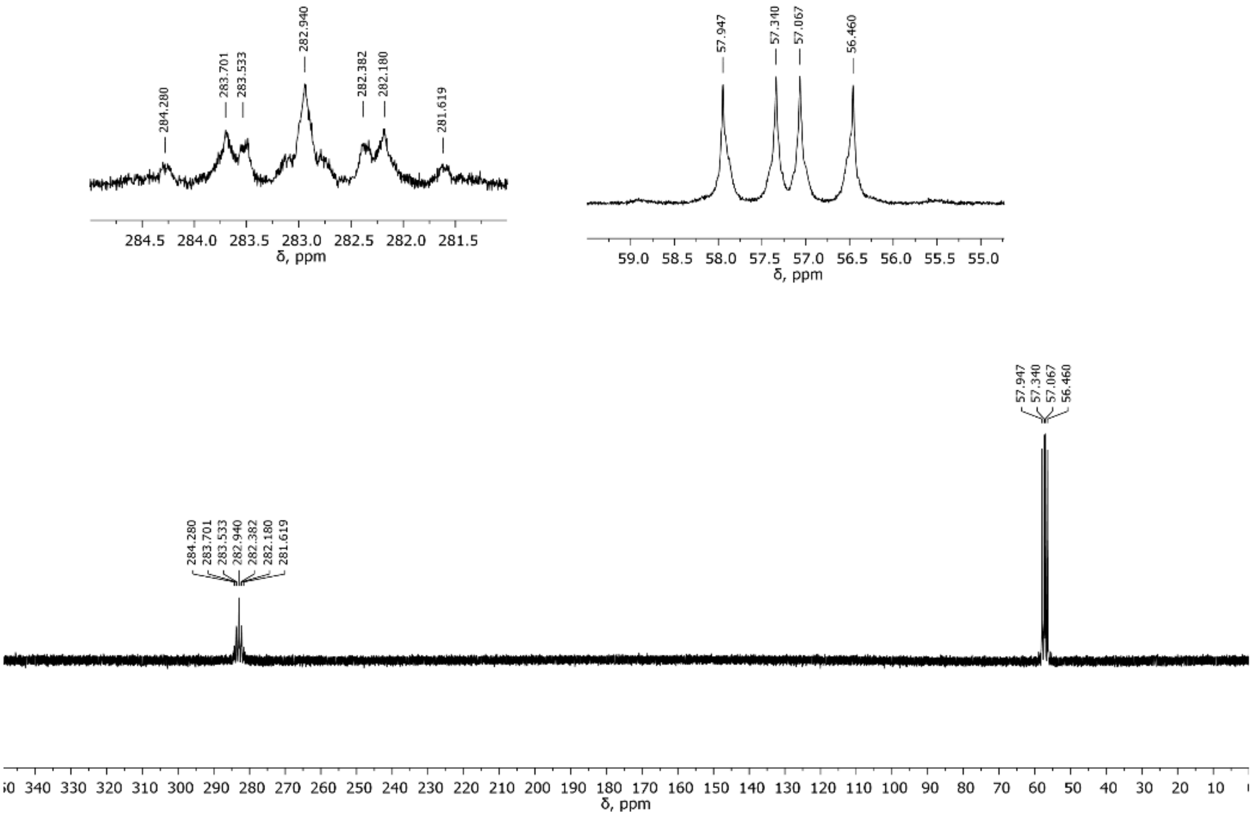

Figure S11. $^{31}P\{^{1}H\}$ NMR spectrum (202 MHz, C_6D_6) of **4**.

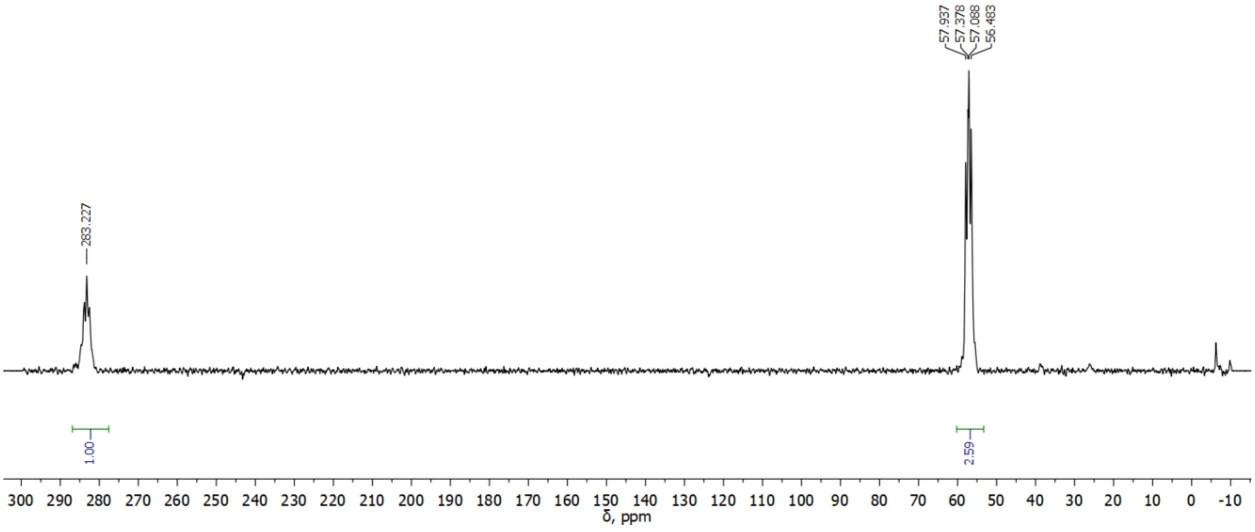

Figure S12. $^{31}P\{^{1}H\}$ NMR spectrum (202 MHz, THF) of **4** before workup.

Synthesis of 5. To a J. Young NMR tube was added **4** (32 mg, 0.032 mmol) and dissolved in $CDCl_3$ (0.7 mL). Over the course of four hours, the solution gradually changed color from dark-brown to dark-blue. Once the reaction was complete, as determined by NMR analysis, the solution was transferred into a 20 mL scintillation vial and ca. 5 mL pentane (the product **5** is minimally soluble in pentane) was added, and the solution was left in a -38 °C freezer for 11 h to afford blue crystals. The mother liquor was removed and the crystals were dissolved in benzene and frozen. The product was freeze-dried to afford a fine blue powder (17 mg, 50% yield). Single crystals suitable for XRD were grown by dissolving the product in toluene and placing the solution in a small vial. The small vial was then placed in a larger vial with pentane and pentane vapor was allowed to diffuse over the course of 3 days at room temperature. ^{1}H NMR (500 MHz, $CDCl_3$): δ 6.63 (d, J = 3.2 Hz, pyrrole ring, 1H), 6.45 (t, *J* = 3 Hz, pyrrole ring, 1H), 6.41 (s, pyrrole ring, 1H), 6.40 (s, pyrrole ring, 1H), 6.28 (brs, pyrrole ring, 1H), 6.18 (brs, pyrrole ring, 1H), 2.75 – 2.86 (m, $CHMe_2$ with *H* italic: C*H*Me_2, 2H), 2.62 (m, C*H*Me_2, 1H), 1.92 (C*H*Me_2, 1H), 1.52 (dd, *J* = 16.0, 7.3 Hz, CHMe_2, 3H), 1.42 (dd, *J* = 12.9, 7.3 Hz, CHMe_2, 3H), 1.16 – 1.28 (m, CHMe_2, 12H), 1.06 (dd, J = 16.0, 7.3 Hz, CHMe_2, 3H), 0.97 (dd, J = 18.8, 7.0 Hz, CHMe_2, 3H). $^{13}C\{^{1}H\}$ NMR (125.75 MHz, $CDCl_3$): δ 139.2 – 139.8 (m), 127.1 (d, J_{C-P} = 8.9 Hz), 124.5, 119.0 (d, J_{C-P} = 11.34 Hz), 118.2, 117.9 (d, J_{C-P} = 4.5 Hz), 114.5 (d, J_{C-P} = 9.2 Hz), 31.6 (m), 31.5 (m), 31.1 (vt, J_{C-P} = 6.0 Hz), 30.6 (m), 25.4, 25.2, 24.4 (m), 21.2 (d, J_{C-P} = 4.2 Hz), 19.7, 19.5, 19.3 (m), 19.0 (m), 18.9 (m). $^{31}P\{^{1}H\}$ NMR (202 MHz, $CDCl_3$): δ 218.9 (m), 38.1 (m), 34.0. Elem. Anal. Found (Calculated) for $C_{40}H_{68}N_4P_6Cl_2Rh_2$ C, 45.07 (45.00); H, 6.50 (6.42); N, 5.00 (5.25)

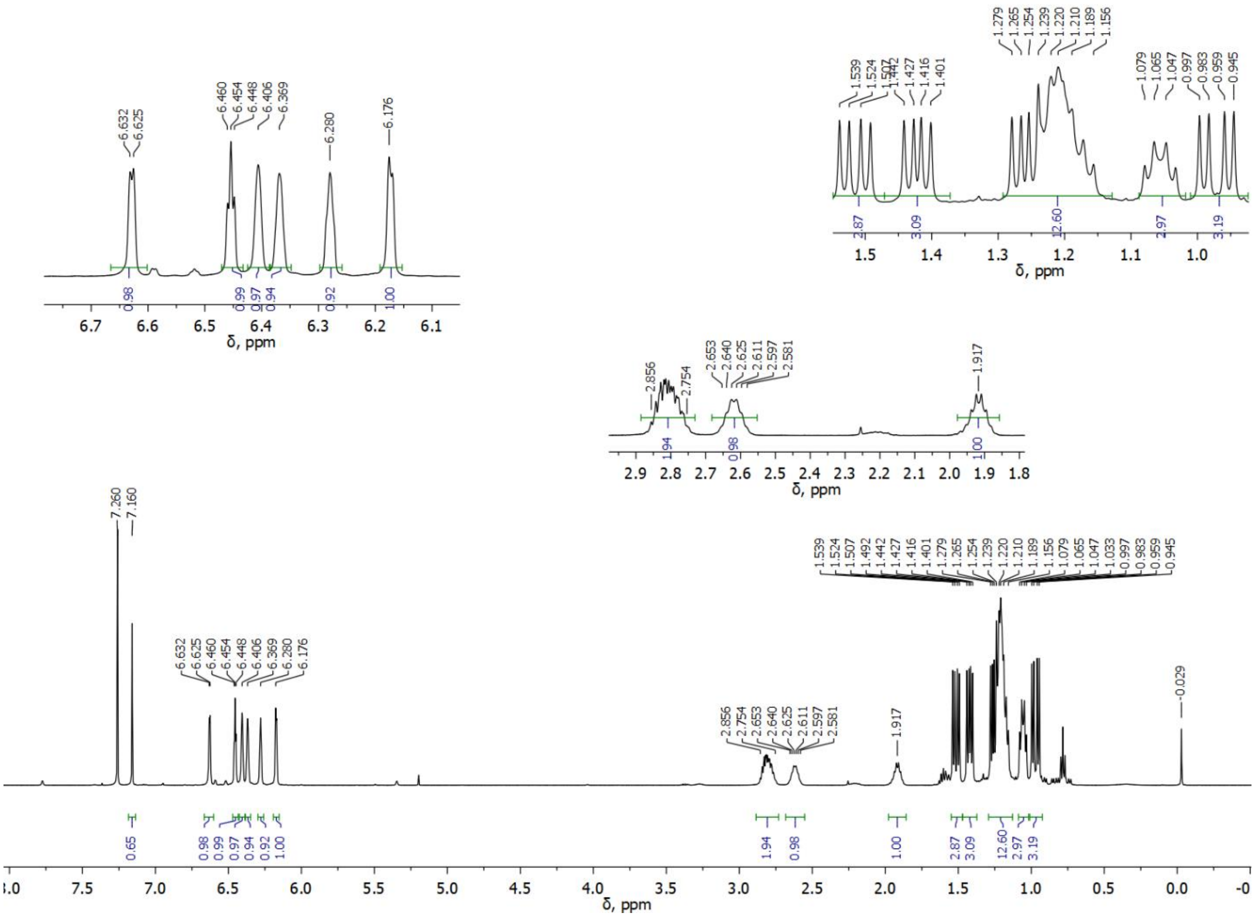

Figure S13. ^{1}H NMR spectrum (500 MHz, $CDCl_3$) of **5**.

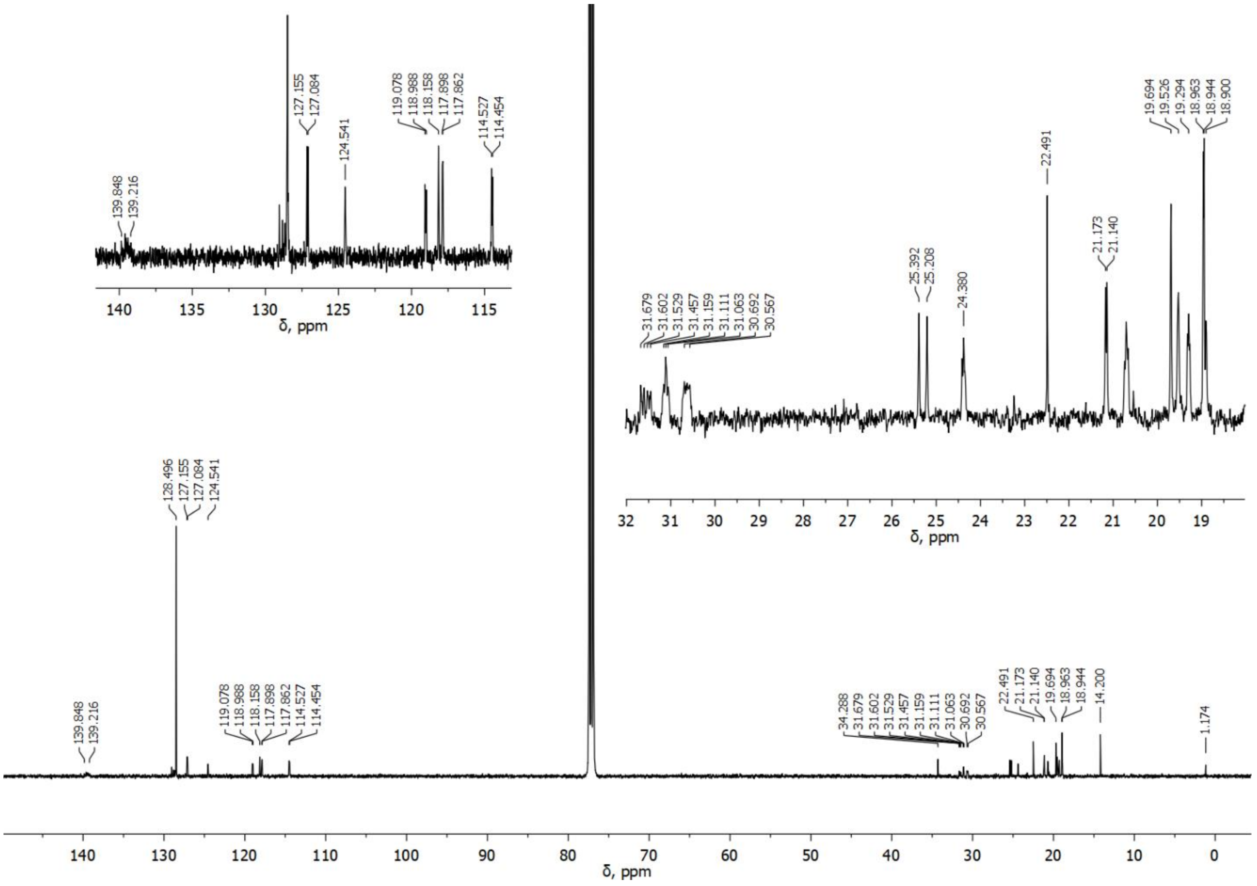

Figure S14. $^{13}C\{^{1}H\}$ NMR spectrum (126 MHz, $CDCl_3$) of **5**.

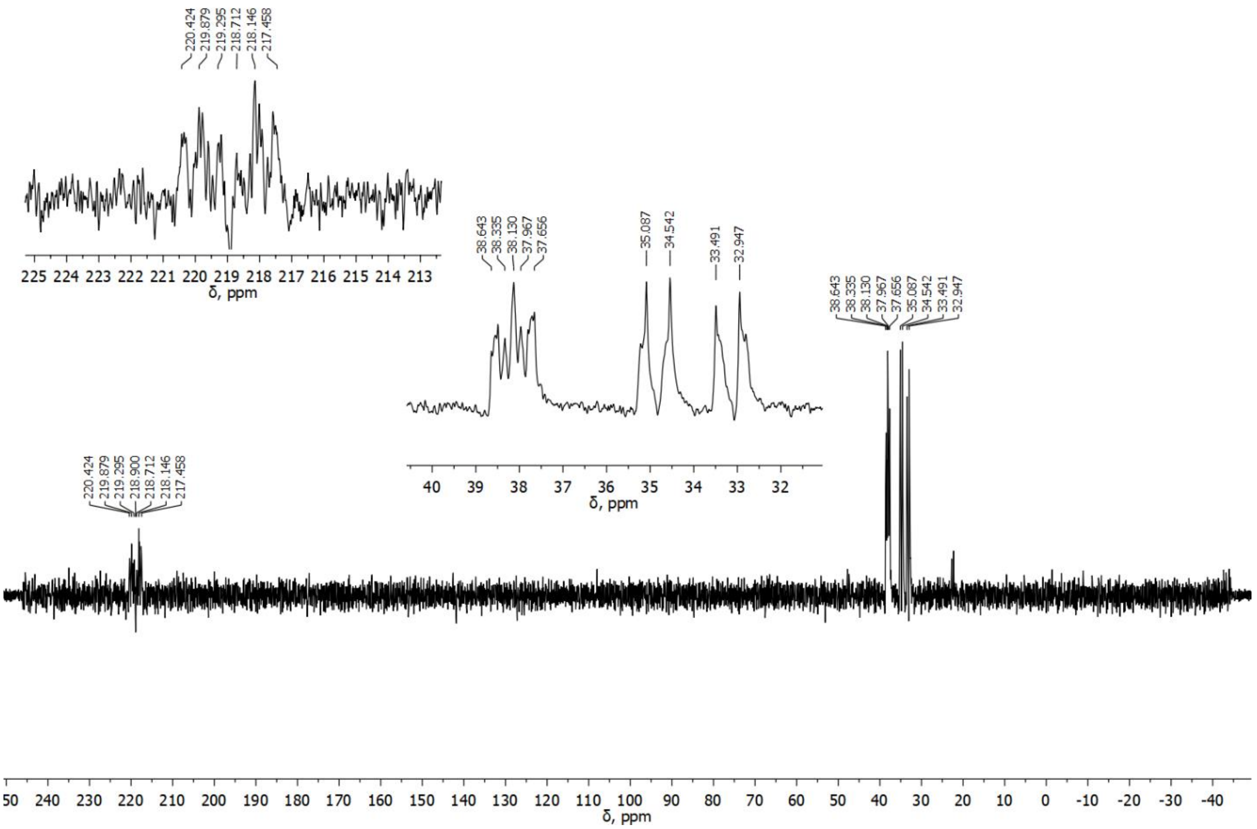

Figure S15. $^{31}P\{^{1}H\}$ NMR spectrum (202 MHz, $CDCl_3$) of **5**.

Attempted reaction of 4 with bromobenzene. A J. Young NMR tube was charged with **4** (48 mg, 0.048 mmol) and PhBr (10 µL, 0.095 mmol) followed by 0.7 mL C_6D_6. The reaction was heated in a 75 °C oil bath for 20 h. No change was observed by ^{1}H or $^{31}P\{^{1}H\}$ NMR spectroscopy.

Synthesis of 6. In a J. Young NMR tube, **4** (34 mg, 0.034 mmol) was dissolved in THF (0.7 mL) and degassed via three freeze-pump-thaw cycles. The solution was then placed under an atmosphere of CO and swirled until the color of the solution became a clear red. The volatiles were removed under vacuum and 1 mL of acetonitrile was added, followed by a minimal amount of toluene (ca. 50 µL) to create a homogeneous solution. The solution was placed in a -38 °C freezer for 24 h to afford dark brown crystals. The mother liquor was then removed and the crystals

dissolved in benzene. The product was freeze-dried to afford a brown powder (17 mg, 50% yield). Single crystals were grown by dissolving **6** in pentane and then placing the loosely capped vial in a -38 °C freezer for 1 week which afforded single crystals suitable for XRD. IR: 1943 cm^{-1}. ^{1}H NMR (400 MHz, C_6D_6): δ 6.63 (s, pyrrole ring, 4H), 6.48 (t, *J* = 3.7 Hz, pyrrole ring, 4 H), 6.32 (dd, *J* = 3.2, 1.2 Hz, pyrrole ring, 4H), 2.20 (m, C*H*Me_2, 4H), 2.07 (m, C*H*Me_2, 4H), 1.03 – 1.16 (m, CH*Me*$_2$, 48H). $^{13}C\{^{1}H\}$ NMR (100 MHz, C_6D_6): δ 205.4 (br), 136.6 (m), 126.2, 117.0, 113.9, 28.8, 27.9, 19.9, 19.7, 18.9, 18.7. $^{31}P\{^{1}H\}$ NMR (202 MHz, C_6D_6, 25 °C): δ 49.10-35.15 (overlapping signals, 6P). $^{31}P\{^{1}H\}$ NMR (202 MHz, C_7D_8, -55 °C): δ 54.68 (m, 2P), 46.00 (m, 2P), 30.14 (m, 2P). $^{31}P\{^{1}H\}$ NMR (202 MHz, C_7D_8, 80 °C): δ 43.86 (m, 2P), 42.08 (m, 4P). Elem. Anal. Found (Calculated) for $C_{42}H_{68}N_4P_6O_2Rh_2$ C, 47.92 (48.27); H, 6.51 (6.34); N, 4.92 (5.32).

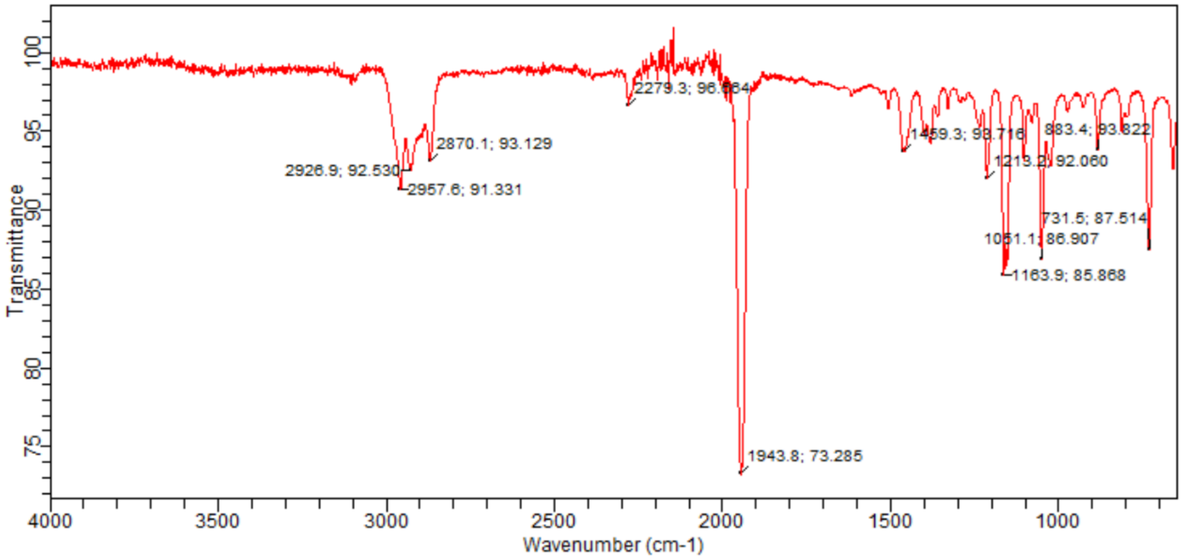

Figure S16. FT-IR Spectrum of **6** in C_6D_6.

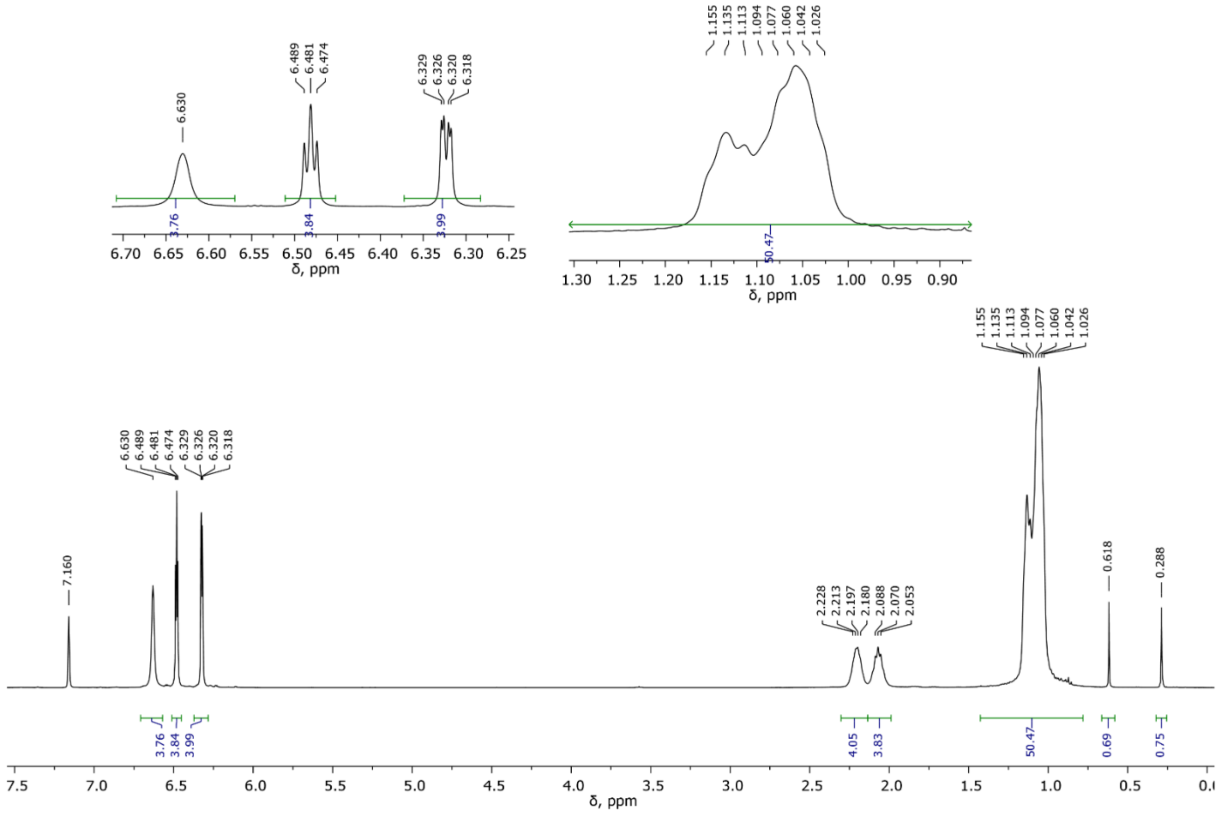

Figure S17. ^{1}H NMR spectrum (400 MHz, C_6D_6) of **6**. Residual CH_3CN (0.62 ppm) and silicon grease (0.29) visible.

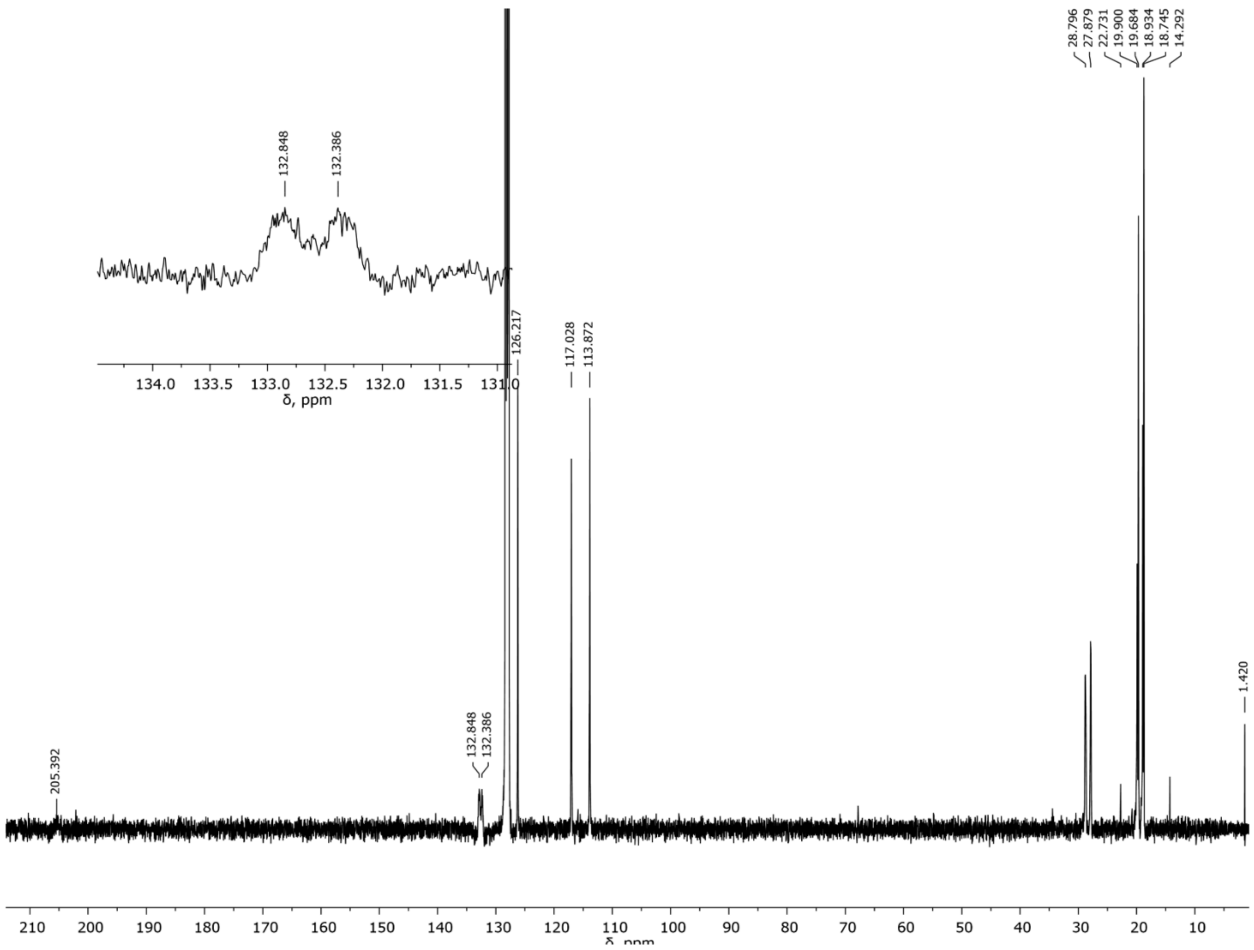

Figure S18. $^{13}C\{^1H\}$ NMR spectrum (100 MHz, C_6D_6) of **6**. Silicon grease (1.4 ppm) and pentane (22.7, 14.3 ppm) visible.

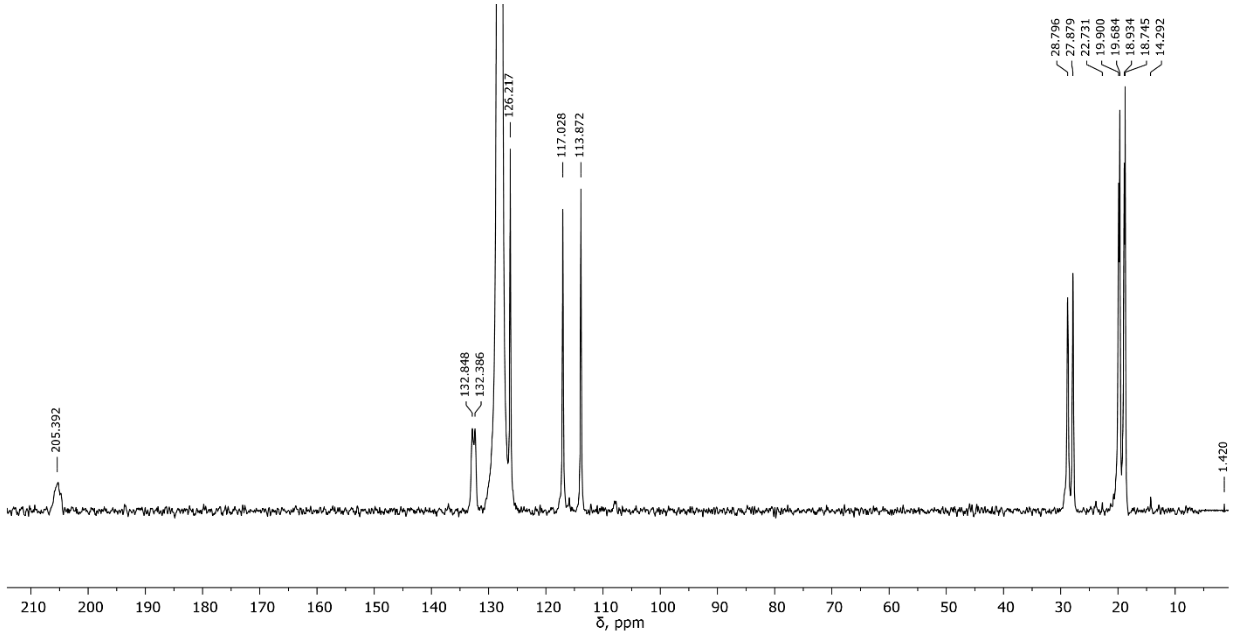

Figure S19. $^{13}C\{^1H\}$ NMR spectrum (100 MHz, C_6D_6) of **6** with applied line broadening.

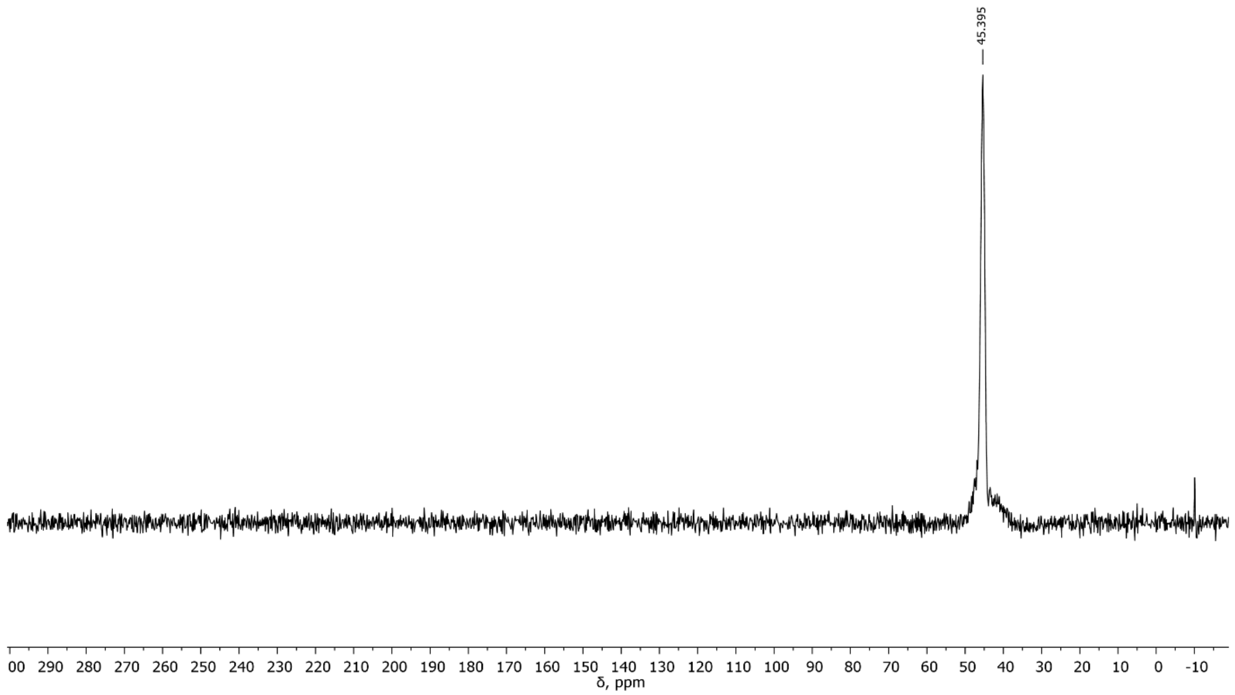

Figure S20. $^{31}P\{^1H\}$ NMR spectrum (202 MHz, C_6D_6) of **6**.

Variable temperature $^{31}P\{^1H\}$ NMR study of 6 under 1 atm CO. A J. Young NMR tube was charged with 18.0 mg **6** (0.017 mmol) and dissolved in 0.5 mL C_7D_8, and the solution was degassed via 3 cycles of freeze-pump-thaw before refilling with 1 atm CO. Variable temperature NMR

spectra were recorded observing $^{31}P\{^1H\}$ on a Bruker Avance Neo 500 spectrometer from 25 °C to -75 °C. The probe and sample were returned to 25 °C, and spectra were recorded from 25 °C to 95 °C, before the probe and sample were returned to 25 °C.

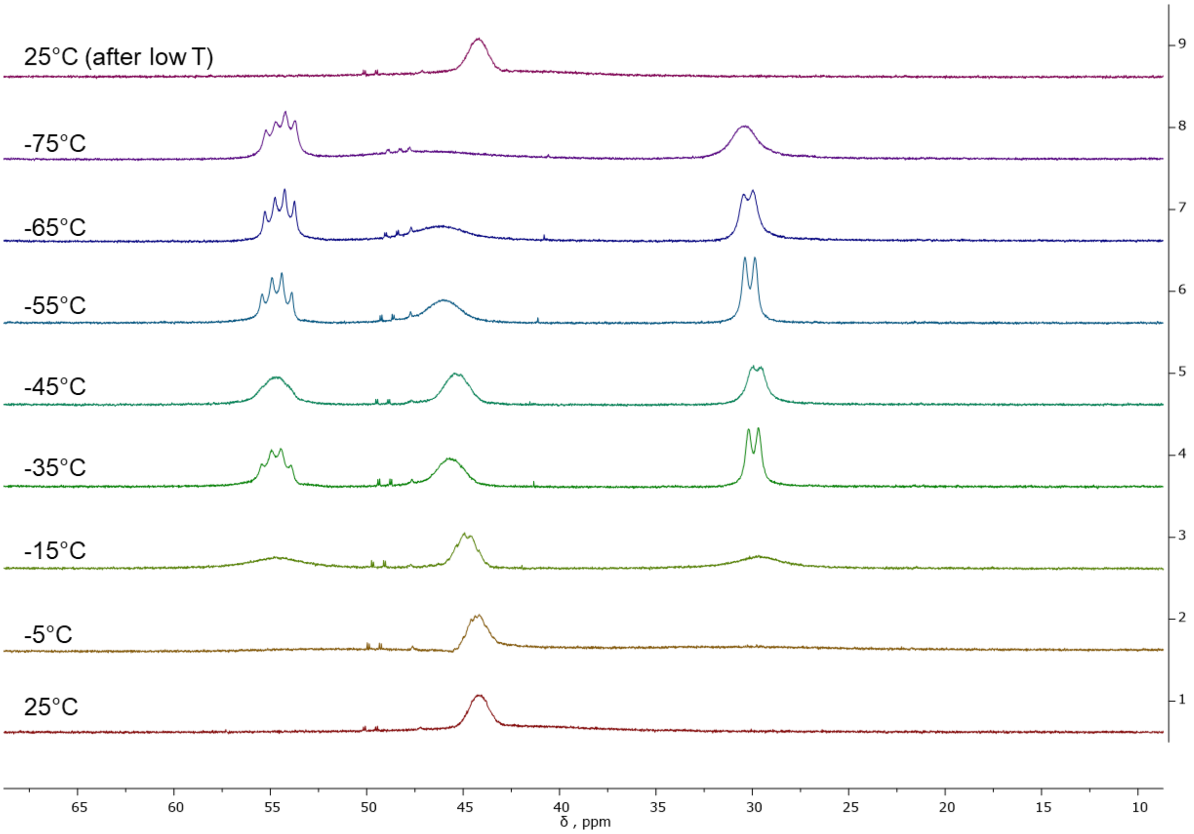

Figure S21. Low temperature $^{31}P\{^1H\}$ NMR study (202 MHz, C_7D_8) of **6** under 1 atm CO.

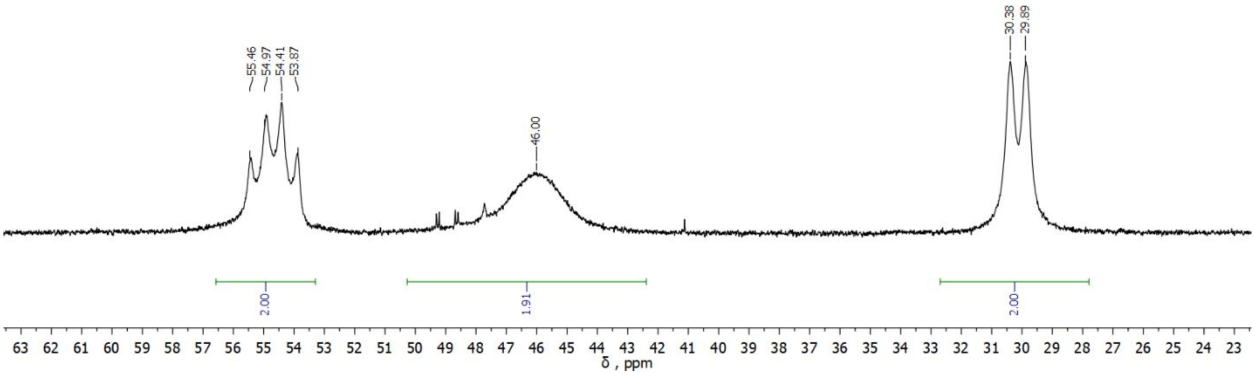

Figure S22. $^{31}P\{^1H\}$ NMR spectrum (202 MHz, C_7D_8, -55 °C) of **6** under 1 atm CO.

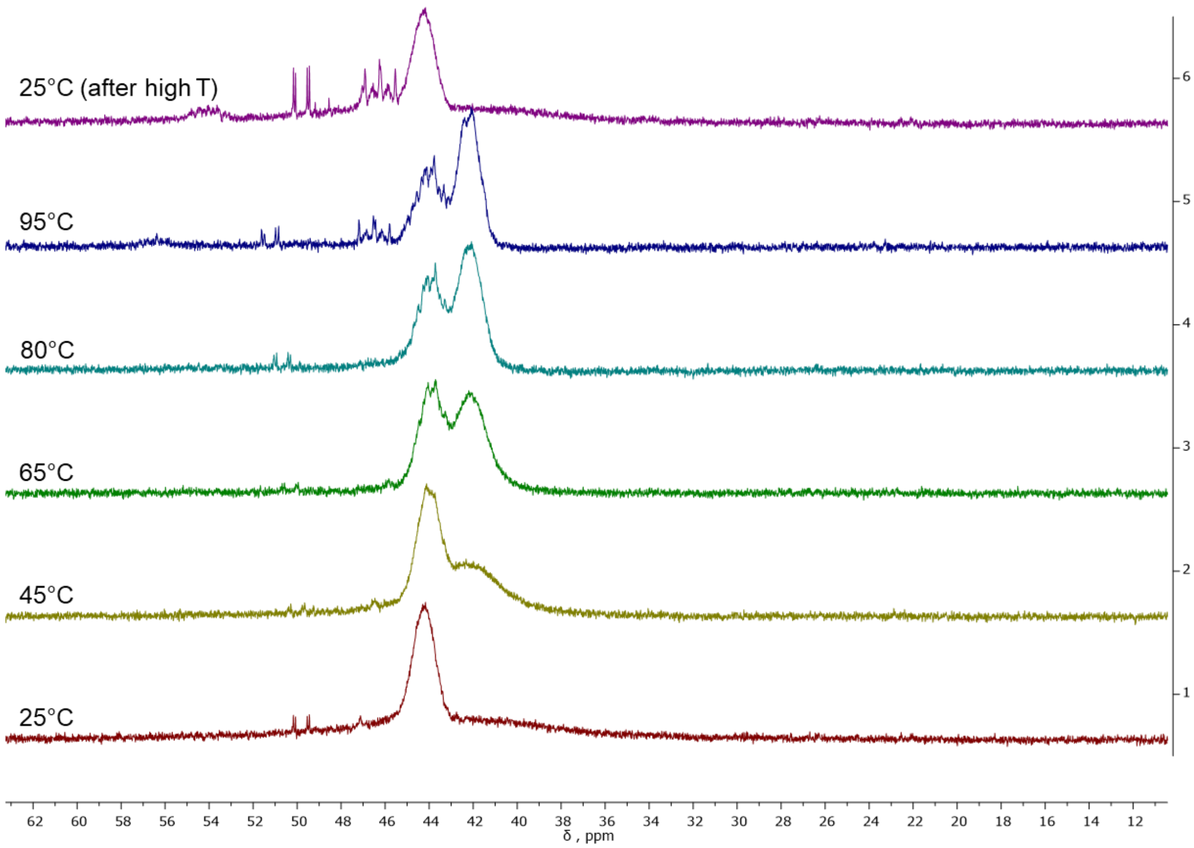

Figure S23. High temperature $^{31}P\{^{1}H\}$ NMR study (202 MHz, C_7D_8) of **6** under 1 atm CO.

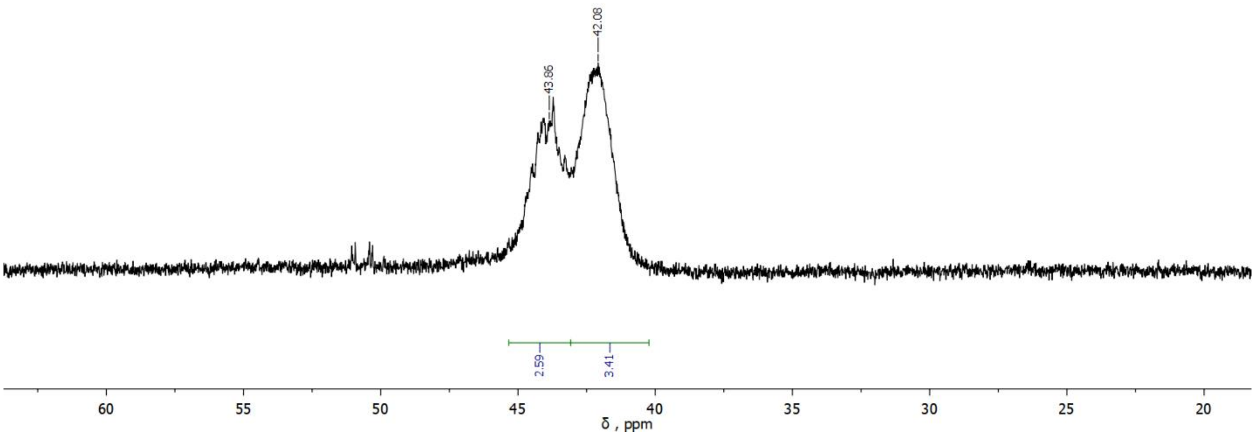

Figure S24. $^{31}P\{^{1}H\}$ NMR study (202 MHz, C_7D_8, 80 C) of **6** under 1 atm CO.

Addition of PPh_3 Integration Standard to 6. A J. Young NMR tube was charged with 17 mg (0.0188 mmol) and 0.7 mL C_7D_8. The tube was degassed and refilled with 1 atm CO followed by vigorous shaking before stirring. After 15 minutes of stirring, **6** was observed as the sole product

by $^{31}P\{^{1}H\}$ NMR. To the J. Young tube was added 15 mg PPh_3 (0.0564 mmol) under argon atmosphere. By $^{31}P\{^{1}H\}$ NMR, the signals for **4** and PPh_3 integrated 6:3 (**Figure S24**).

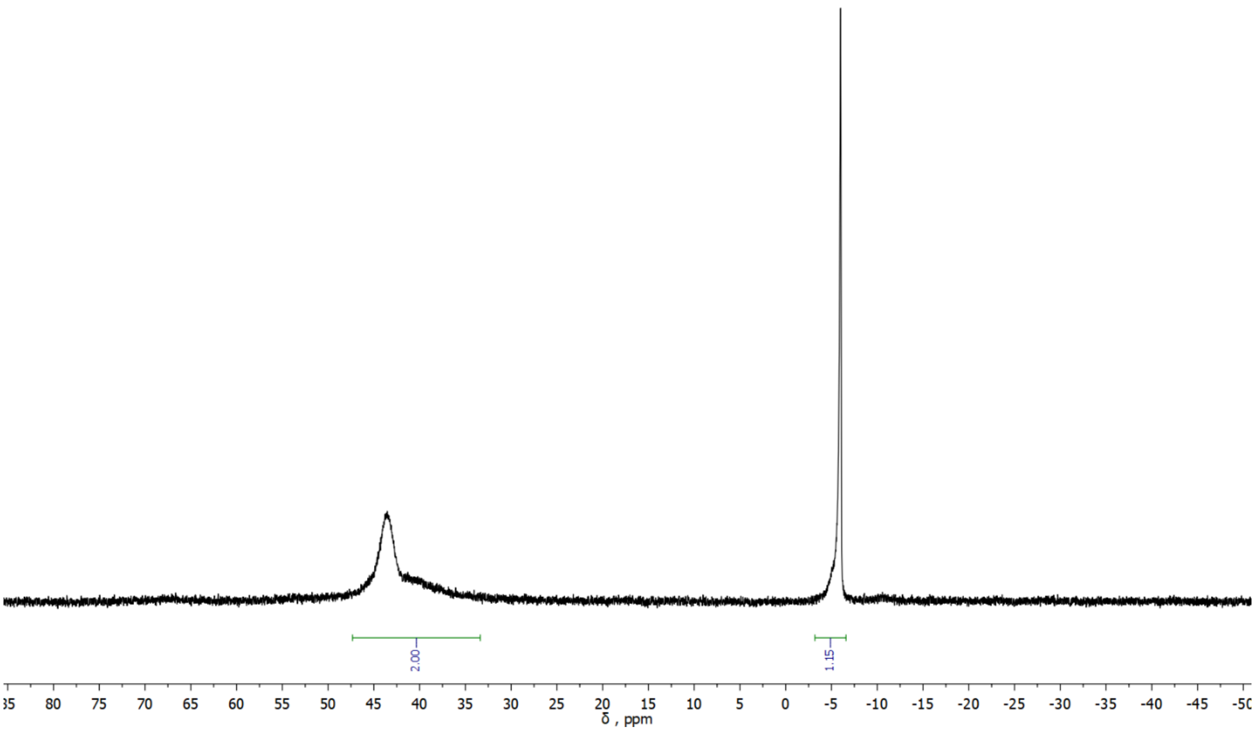

Figure S25. $^{31}P\{^{1}H\}$ NMR spectrum (162 MHz, C_7D_8) of **6** with 3 equivalents of PPh_3.

III. X-ray Structural Determination Details

X-ray data collection, solution, and refinement for 3a (CCDC: 1911259). A Leica MZ 75 microscope was used to identify a suitable yellow block with very well defined faces with dimensions (max, intermediate, and min) 0.214 x 0.182 x 0.126 mm^3 from a representative sample of crystals of the same habit. The crystal mounted on a nylon loop was then placed in a cold nitrogen stream (Oxford) maintained at 110 K. A BRUKER APEX 2 Duo X-ray (three-circle) diffractometer was employed for crystal screening, unit cell determination, and data collection. The goniometer was controlled using the APEX3 software suite,[3] v2017.3-0.1 The sample was optically centered with the aid of a video camera such that no translations were observed as the crystal was rotated through all positions. The detector was set at 6.0 cm from the crystal sample (APEX2, 512x512 pixel). The X-ray radiation employed was generated from a Mo sealed X-ray tube (K_α = 0.71073Å with a potential of 40 kV and a current of 40 mA).

45 data frames were taken at widths of 1.0°. These reflections were used in the auto-indexing procedure to determine the unit cell using Cell_Now,[4] which indicated two major domains. A suitable cell was found and refined by nonlinear least squares and Bravais lattice procedures. The unit cell was verified by examination of the *h k l* overlays on several frames of data. No super-cell or erroneous reflections were observed. After careful examination of the unit cell, an extended data collection procedure (8 sets) was initiated using omega and phi scans. Integrated intensity information for each reflection was obtained by reduction of the data frames with the program APEX3,[3] including both the domains. The integration method employed a three dimensional profiling algorithm and all data were corrected for Lorentz and polarization factors, as well as for crystal decay effects. Finally, the data was merged and scaled to produce a suitable data set. The absorption correction program TWINABS[5] was employed to correct the data for absorption

effects, as well as to generate *hkl* files. Systematic reflection conditions and statistical tests of the data suggested the space group *P*-1. A solution was obtained readily using XT/XS in APEX2.[3,6] Fluorobenzene was found solvated with a disorder in Fluorine position, which was modeled. Hydrogen atoms were placed in idealized positions and were set riding on the respective parent atoms. All non-hydrogen atoms were refined with anisotropic thermal parameters. Absence of additional symmetry and voids were confirmed using PLATON (ADDSYM).[7] The structure was refined (weighted least squares refinement on *F2*) to convergence.[6,8]

X-ray data collection, solution, and refinement for 4 (CCDC: 1911260)**.** A suitable brown block with very well defined faces with dimensions (max, intermediate, and min) 0.242 x 0.089 x 0.03 mm^3 was picked from a representative sample of crystals of the same habit. The crystal mounted on a nylon loop was then placed in a cold nitrogen stream (Oxford) maintained at 100 K. A BRUKER Venture X-ray (kappa geometry) diffractometer was employed for crystal screening, unit cell determination, and data collection. The goniometer was controlled using the APEX3 software suite.[3] The sample was optically centered with the aid of a video camera such that no translations were observed as the crystal was rotated through all positions. The X-ray radiation employed was generated from a Cu-Iμs X-ray tube (K_α = 1.5418Å with a potential of 50 kV and a current of 1.0mA). 45 data frames were taken at widths of 1°. These reflections were used to determine the unit cell using Cell_Now,[4] which indicated two component twin domains. The unit cell was verified by examination of the *h k l* overlays on several frames of data. No super-cell or erroneous reflections were observed. After careful examination of the unit cell, an extended data collection procedure (19 sets) was initiated using omega and phi scans. Integrated intensity information for each reflection was obtained by reduction of the data frames with the program

APEX3,[3] including the two domains. The integration method employed a three dimensional profiling algorithm and all data were corrected for Lorentz and polarization factors, as well as for crystal decay effects. Finally the data was merged and scaled to produce a suitable data set. The absorption correction program TWINABS2[5] was employed to correct the data for absorption effects, as well as to generate twin4.hkl, and twin5.hkl files. While the former was used for structure solution, the latter was used for final least squares refinement. Systematic reflection conditions and statistical tests of the data suggested the space group *P*21. A solution was obtained readily (Z=4; Z'= 2) using XT/XS in APEX3.[3,6] Hydrogen atoms were placed in idealized positions and were set riding on the respective parent atoms. All non-hydrogen atoms were refined with anisotropic thermal parameters. Absence of additional symmetry and voids were confirmed using PLATON (ADDSYM).[7] The structure was refined (weighted least squares refinement on *F2*) to convergence.[3,8]

X-ray data collection, solution, and refinement for 5 (CCDC: 1911261)**.**

A Leica MZ 75 microscope was used to identify a suitable brown block with very well defined faces with dimensions (max, intermediate, and min) 0.357 x 0.208 x 0.086 mm^3 from a representative sample of crystals of the same habit. The crystal mounted on a nylon loop was then placed in a cold nitrogen stream (Oxford) maintained at 110 K.

A BRUKER APEX 2 Duo X-ray (three-circle) diffractometer was employed for crystal screening, unit cell determination, and data collection. The goniometer was controlled using the APEX3 software suite,[3] v2017.3-0.1. The sample was optically centered with the aid of a video camera such that no translations were observed as the crystal was rotated through all positions. The detector was set at 6.0 cm from the crystal sample (APEX2, 512x512 pixel). The X-ray radiation

employed was generated from a Mo sealed X-ray tube (Kα = 0.71073Å with a potential of 40 kV and a current of 40 mA).

45 data frames were taken at widths of 1.0°. These reflections were used in the auto-indexing procedure to determine the unit cell. A suitable cell was found and refined by nonlinear least squares and Bravais lattice procedures. The unit cell was verified by examination of the *h k l* overlays on several frames of data. No super-cell or erroneous reflections were observed.

After careful examination of the unit cell, an extended data collection procedure (7 sets) was initiated using omega and phi scans.

Data Reduction, Structure Solution, and Refinement

Integrated intensity information for each reflection was obtained by reduction of the data frames with the program APEX3.[3] The integration method employed a three dimensional profiling algorithm and all data were corrected for Lorentz and polarization factors, as well as for crystal decay effects. Finally the data was merged and scaled to produce a suitable data set. The absorption correction program SADABS[9] was employed to correct the data for absorption effects. Systematic reflection conditions and statistical tests of the data suggested the space group *P*21212. A solution was obtained readily (Z=2; Z'=0.5) using XT/XS in APEX2.[3,6] Hydrogen atoms were placed in idealized positions and were set riding on the respective parent atoms. All non-hydrogen atoms were refined with anisotropic thermal parameters. Absence of additional symmetry and voids were confirmed using PLATON (ADDSYM).[7] The structure was refined (weighted least squares refinement on *F2*) to convergence.[7,8]

X-ray data collection, solution, and refinement for 6 (CCDC: 1911262)**.**

A Leica MZ 75 microscope was used to identify a suitable pink block with very well defined faces with dimensions (max, intermediate, and min) 0.723 x 0.343 x 0.12 mm^3 from a representative sample of crystals of the same habit. The crystal mounted on a nylon loop was then placed in a cold nitrogen stream (Oxford) maintained at 110 K.

A BRUKER APEX 2 Duo X-ray (three-circle) diffractometer was employed for crystal screening, unit cell determination, and data collection. The goniometer was controlled using the APEX3 software suite,[3] v2017.3-0. The sample was optically centered with the aid of a video camera such that no translations were observed as the crystal was rotated through all positions. The detector was set at 6.0 cm from the crystal sample (APEX2, 512x512 pixel). The X-ray radiation employed was generated from a Mo sealed X-ray tube (Kα = 0.71073Å with a potential of 40 kV and a current of 40 mA).

45 data frames were taken at widths of 1.0°. These reflections were used in the auto-indexing procedure to determine the unit cell. A suitable cell was found and refined by nonlinear least squares and Bravais lattice procedures. The unit cell was verified by examination of the *h k l* overlays on several frames of data. No super-cell or erroneous reflections were observed.

After careful examination of the unit cell, an extended data collection procedure (8 sets) was initiated using omega and phi scans.

Data Reduction, Structure Solution, and Refinement

Integrated intensity information for each reflection was obtained by reduction of the data frames with the program APEX3.[3] The integration method employed a three dimensional profiling algorithm and all data were corrected for Lorentz and polarization factors, as well as for crystal decay effects. Finally the data was merged and scaled to produce a suitable data set. The absorption correction program SADABS[9] was employed to correct the data for absorption effects. Systematic reflection conditions and statistical tests of the data suggested the space group *P*21/*n*. A solution was obtained readily using XT/XS in APEX2.[3,6] A molecule of pentane was found solvated. Hydrogen atoms were placed in idealized positions and were set riding on the respective parent atoms. All non-hydrogen atoms were refined with anisotropic thermal parameters. Absence of additional symmetry and voids were confirmed using PLATON (ADDSYM).[7] The structure was refined (weighted least squares refinement on *F2*) to convergence.[3,8]

IV. Computational methods.

The Gaussian 09 suite of programs[10] was used for the ab initio electronic structure calculations. All structures were fully optimized using the M06[11] functional in the gas phase, and harmonic vibrational frequency calculations were performed to ensure that either a minimum was obtained. The Los Alamos basis set and the associated effective core potential (ECP) was used for Rh atom, and an all-electron 6-31G(d) basis set was used for all the other atoms.

V. SI References

[1] Herdé, J. L.; Lambert, J. C.; Senoff, C. V. Cyclooctene and 1,5-Cyclooctadiene Complexes of Iridium(I). *Inorg. Synth.* **1974**, *15*, 18–20.

[2] Lai, Q.; Cosio, M. N.; Ozerov, O. V. Ni Complexes of an Alane/Tris(phosphine) Ligand Built Around a Strongly Lewis Acidic Tris(N-pyrrolyl)aluminum. *Chem. Commun.* **2020**, *56*, 14845-14848.

[3] *APEX3: Program for Data Collection on Area Detectors*; Bruker AXS Inc., Madison, WI, USA, 2016.

[4] Sheldrick, G. M. *Cell_Now, Program for Obtaining Unit Cell Constants from Single Crystal Data*, version 2008/1; University of Göttingen, Germany, 2008.

[5] G. M. Sheldrick, *TWINABS: Program for Absorption Correction of Area Detector Frames*; Bruker AXS Inc., Madison, WI, USA.

[6] (a) Sheldrick, G. M. A Short History of *SHELX*. *Acta Crystallogr., Sect. A: Found. Crystallogr.* **2008**, *64*, 112−122. (b) Sheldrick, G. M. *SHELXT* – Integrated Space-Group and Crystal-Structure Determination. *Acta Crystallogr., Sect. A: Found. Crystallogr.* **2015**, *71*, 3−8. (c) Sheldrick, G. M. Crystal Structure Refinement with *SHELXL*. *Acta Crystallogr., Sect. C: Struct. Chem.* **2015**, *71*, 3−8. (d) *XT*, *XS*; Bruker AXS Inc.: Madison, WI, USA.

[7] Spek, A. L. Single-Crystal Structure Validation with the Program *PLATON*. *J. Appl. Cryst.* **2003**, *36*, 7-13.

[8] Dolomanov, O. V, Bourhis, L. J., Gildea, R. J., Howard, J. A. K., and Puschmann, H. *OLEX2*: A Complete Structure Solution, Refinement and Analysis Program, *J. Appl. Cryst.* **2009**, *42*, 339-341.

[9] Sheldrick, G.M. *SADABS: Program for Absorption Correction of Area Detector Frames*; Bruker AXS Inc.: Madison, WI, USA, 2008.

[10] Frisch, M. J.; Trucks, G. W.; Schlegel, H. B.; Scuseria, G. E.; Robb, M. A.; Cheeseman, J. R.; Scalmani, G.; Barone, V.; Mennucci, B.; Petersson, G. A.; Nakatsuji, H.; Caricato, M.; Li, X.; Hratchian, H. P.; Izmaylov, A. F.; Bloino, J.; Zheng, G.; Sonnenberg, J. L.; Hada, M.; Ehara, M.; Toyota, K.; Fukuda, R.; Hasegawa, J.; Ishida, M.; Nakajima, T.; Honda, Y.; Kitao, O.;

Nakai, H.; Vreven, T.; Montgomery. Jr., J. A.; Peralta, J. E.; Ogliaro, F.; Bearpark, M.; Heyd, J. J.; Brothers, E.; Kudin, K. N.; Staroverov, V. N.; Kobayashi, R.; Normand, J.; Raghavachari, K.; Rendell, A.; Burant, J. C.; Iyengar, S. S.; Tomasi, J.; Cossi, M.; Rega, N.; Millam, N. J.; Klene, M.; Knox, J. E.; Cross, J. B.; Bakken, V.; Adamo, C.; Jaramillo, J.; Gomperts, R.; Stratmann, R. E.; Yazyev, O.; Austin, A. J.; Cammi, R.; Pomelli, C.; Ochterski, J. W.; Martin, R. L.; Morokuma, K.; Zakrzewski, V. G.; Voth, G. A.; Salvador, P.; Dannenberg, J. J.; Dapprich, S.; Daniels, A. D.; Farkas, Ö.; Foresman, J. B.; Ortiz, J. V.; Cioslowski, J.; Fox, D. J. *Gaussian 09*, Revision D.01; Gaussian, Inc.: Wallingford, CT, 2009.

[11] Zhao, Y.; Truhlar, D. The M06 suite of density functionals for main group thermochemistry, thermochemical kinetics, noncovalent interactions, excited states, and transition elements: two new functionals and systematic testing of four M06-class functionals and 12 other functionals. *Theor. Chem. Acc*. **2008**, 120, 215– 241
